# Effect of Elevated CO_2_ on Seed Yield, Essential Oil Metabolism, Nutritive Value, and Biological Activity of *Pimpinella anisum* L. Accessions at Different Seed Maturity Stages

**DOI:** 10.3390/biology10100979

**Published:** 2021-09-29

**Authors:** Mansour A. Balkhyour, Abdelrahim H. A. Hassan, Riyadh F. Halawani, Ahmed Saleh Summan, Hamada AbdElgawad

**Affiliations:** 1 Department of Environmental Science, Faculty of Meteorology, Environment and Arid Land Agriculture, King Abdulaziz University, Jeddah 21589, Saudi Arabia; Rhalawani@kau.edu.sa (R.F.H.); asumman@kau.edu.sa (A.S.S.); 2 Department of Food Safety and Technology, Faculty of Veterinary Medicine, Beni-Suef University, Beni-Suef 62511, Egypt; 3 Centre of Excellence in Environmental Studies, King Abdulaziz University, Jeddah 21589, Saudi Arabia; 4 Department of Botany and Microbiology, Faculty of Science, Beni-Suef University, Beni-Suef 62511, Egypt; hamada.abdelgawad@science.bsu.edu.eg

**Keywords:** aniseed, elevated CO_2_, essential oil metabolism, seed maturity, plant origin

## Abstract

**Simple Summary:**

This study was conducted to investigate whether the positive impact of elevated CO_2_ (eCO_2_) on the chemical composition of aniseed (*Pimpinella anisum* L.) seeds is dependent on seed developmental stages and origin. To this end, we investigated the biochemical changes in eCO_2_-treated aniseed accessions from Tunisia, Syria, Turkey, Morocco, Yemen, and Egypt during three developmental stages (immature, premature, and mature). The highest dry weight percentages and seed yields were recorded for the Egypt and Morocco accessions. eCO_2_ has inducing properties on the nutritive and biological values of aniseeds, yet its effectiveness is related to seed maturity and provenances. For instance, seed maturation increased the nutrients and antioxidant metabolites in most eCO_2_-treated accessions. Conversely, essential oil metabolism was decreased by seed maturation but this effect was significantly reduced by the use of eCO_2_. The enhanced accumulation of bioactive compounds in eCO_2_-treated seeds was accompanied by improved health benefits. In this regard, eCO_2_ induces the antioxidant and hypocholesterolemic activities of aniseeds, particularly at mature stages. Thus, the present study confirms that there are significant interactions between eCO_2_ exposure, aniseed maturity, and origin on the chemical composition and pharmaceutical properties of aniseed.

**Abstract:**

Besides the lack of studies regarding applying elevated CO_2_ (eCO_2_) as a strategy to improve the chemical composition of anise (*Pimpinella anisum* L.) seeds, studies on its interaction with seed developmental stages and origin are very limited. The seed yield, chemical composition, and biological activity of 6 aniseed accessions (Egypt, Tunisia, Syria, Turkey, Yemen, and Morocco) were investigated during three developmental stages (immature, premature, and mature) under control and elevated CO_2_ conditions. Mature seeds from all aniseed accessions had significantly higher (*p* < 0.05) dry weight (DW) percentages than premature and immature seeds. The highest DW percentages were recorded in Egypt and Morocco accessions. Seed maturation increased nutrients and antioxidant metabolites in most eCO_2_-treated accessions. In contrast, essential oils were decreased by seed maturation, while eCO_2_ reversed this effect. Essential oil-related precursors (e.g., phenylalanine) and enzyme activities (3-Deoxy-d-arabino-heptulosonate-7-phosphate synthase (DAHPS) and O–methyltransferase) decreased with seed maturity. However, high CO_2_ reduced this impact and further induced the other essential oil-related precursors (shikimic and cinnamic acids). Consequently, eCO_2_ provoked changes in the antioxidant and hypocholesterolemic activities of aniseeds, particularly at mature stages. Overall, eCO_2_ application, as an efficient way to improve aniseed growth, essential oil metabolism, and chemical composition, was affected by seed maturation and origin. Future studies of eCO_2_-treated aniseeds as a nutraceutical and pharmaceutical product are suggested.

## 1. Introduction

The seeds of aromatic plants are eaten raw or used in foods for their flavor and aroma in cooked and raw foods. For instance, they are used as a food flavoring in soups, poultry, pickles, sweets, chewing gum, and salad [1]. Among aromatic plants, anise (*Pimpinella anisum* L.) seeds are widely used to flavor dishes, drinks, and candies. For example, they are used in seafood dishes and as a breath sweetener and digestive aid [2]. The essential oils of anise seeds are complex mixtures of volatile oils, terpene derivatives, and ordinarily terpenes. In addition, their chemical composition has been well studied [3]. They contain about 4% of essential oil, whereas (E)-Anethole represents 90% of these essential oils. Other common components include stragol, anisaldehyde, γ-himachalene, eisoeugenol, anisol, p-anisic acid and acetoanisol [4]. Anise essential oil is widely used as a flavoring, as well as in medicines and perfumery. The essential oil possesses antioxidant and antimicrobial properties, and it is also used as an appetizer, carminative and sedative agent [2]. In addition to essential oil, the seeds of anise are also rich in antioxidants, such as phenolic acids and flavonoids; it has been used as a nutritional food and traditional medicinal plant for centuries [5].

The chemical compositions of aromatic seeds are indeed affected by the genotype, the ecological conditions, and developmental seed stages [5,6]. The phytochemicals are involved in plant adaptation to environmental conditions; thus, they can be affected by external environmental factors. For example, the essential oil content and its composition are significantly affected by changing weather conditions, including temperature and light levels, particularly during the development of anise seed [7]. In this aspect, manipulating growth conditions is widely used to improve the biomass accumulation and chemical composition and, consequently, the nutritive values of several crop plants [5]. Among these growth conditions, eCO_2_ could significantly modulate chemistry and the nutritional values of herbal seeds [8]. As a substrate for photosynthesis, enriched atmospheric CO_2_ could improve the photosynthetic carbon assimilation, mainly in C_3_ plants, and therefore supply the metabolic energy required for bioactive metabolite production [9]. The improved levels of primary and secondary active metabolites, such as essential oil, essential amino acids, phenolic acid, and flavonoids are involved in improving the nutritive values of several herbal and crop plants. In this context, enriched atmospheric CO_2_ increased the accumulation of bioactive metabolites in plant tissues and seeds [9]. These enhanced levels of bioactive metabolites, shown in fenugreek seeds, were accompanied by improved nutritive properties, including antioxidant capacity, antimicrobial activity, anti-lipid peroxidation, and anti-cholesterol potential [8].

Several studies also indicated the effect of provenance on the phytochemicals of aromatic seeds [5,6]. The study of Bettaieb Rebey et al. [5] reported a variation in the chemical composition and antioxidant activity of four aniseed populations, whereas the highest yield was achieved at full maturity in all accessions, particularly in the Tunisia accession. Moreover, the chemical composition is significantly varied during the seed maturation of *Apiaceae* seeds [5,10]. For example, the increase in essential oil accumulation was reported at the waxy stage of *Apiaceae* seeds, as compared to the ripening stage [11].

Besides the lack of studies regarding the impact of environmental change (eCO_2_) on aniseed chemical composition and biological activity, studies on the interactive effect of both maturation factors and ecological location on the biochemical composition of aniseed are also limited [12,13]. All this motivated us to investigate the impact of eCO_2_ on the yield, chemical composition, and biological activity of aniseed collected from six different locations and during three maturity stages. Profiling of nutrients, essential oil, and antioxidant metabolites was performed. Moreover, we measured the changes in the precursors and activities of the key enzymes involved in essential oil biosynthesis, as well as the provoked changes in antioxidant and hypocholesterolemic activities. Overall, our study contributed to determining the optimal growth conditions, maturity stage, and provenances for inducing the highest nutrient and essential oil accumulation, thereby improving the quality of aniseeds, which could be of value in the functional food industry.

## 2. Material and Methods

### 2.1. Experimental Setup, Growth Conditions, and Plant Harvests

Healthy and uniform seeds of six anise (*Pimpinella anisum* L.) accession cultivars, i.e., Egypt (var. Baladi), Tunisia (var. Dulce), Syria (var. Ajmer Anise-1), Turkey (var. Gülsüm BOZTAŞ1 Emine BAYRAM1), Yemen (var. Fam), and Morocco (halawa2) were sown in potting mix (Tref EGO substrates, Moerdijk, The Netherlands, 35 × 25 cm pots). About 2.5 kg of loamy soil and organic compost (50:50%) were added to pots and the soil water content (SWC) was adjusted to 60%. Five plants were sowed per pot. In total, 96 pots (8 pots per accession and per treatment) were transferred to a controlled-growth cabinet under two climate conditions, viz: (1) ambient CO_2_ (365 ± 37 μmol CO_2_ mole^−1^ air), (2) elevated CO_2_ (eCO_2_) (655 ± 41 μmol CO_2_ mole^−1^ air). The high atmospheric CO_2_ level was continuously monitored and adjusted with a CO_2_ analyzer (WMA-4, PP Systems, Hitchin, UK). The growth conditions of 150 μmol PAR m^−2^ s^−1^, 22/18 °C air temperature and 60% humidity, and 16/8 h day/night photoperiod were adjusted. The plants were watered daily to stabilize the soil water content to 65% of SWC. To avoid cabinet-specific bias, all pots and their CO_2_ treatment were relocated between the two climate cabinets every two weeks. The experiment was repeated twice. The seeds were harvested at three developmental stages, including immature seeds (137 days after sowing), pre-mature seeds (147 days after sowing), and mature seeds (157 days after sowing).

### 2.2. Nutrient Analyses

The total sugar content was measured following the method as described by Nelson [14]. The total lipid content was extracted in chloroform/methanol (2:1, *v*/*v*) and was measured according to Bligh and Dyer [15]. The determination of fibers was performed according to the AOAC method in [16]. The fiber content was precipitated with ethanol and the residue was weighed after washing.

The total protein content was measured using the Folin–Lowry method. The contents of alkaloids and saponins were also measured in the aniseeds. The total polyphenol and flavonoid contents were extracted in 80% ethanol (*v*/*v*) and determined according to the micro-plate method with gallic acid and quercetin, respectively, as standards. More details about these adopted methods are written in Hozzein et al. [8].

### 2.3. Determination of Essential Oil Levels and Metabolism

The aniseeds were air-dried, 15 g of which were used for essential oil extraction. A Clevenger-type instrument was used to steam-distill the dry pieces for 3 h. The essential oils were measured using GC/MS, according to the method described by Okla et al. [17]. The essential oil concentrations were determined as a percentage (%).

The determination of essential oil-related precursors, i.e., phenylalanine, cinnamic acid, and shikimic acid, was carried out using an ultra-performance liquid chromatography system (Waters Acquity UPLC, Milford, Worcester County, MA, USA) coupled with a quadrupole mass spectrometer (Waters Xevo TQ, Milford, Worcester County, MA, USA) equipped with an ESI source, according to the methods that were previously described by Wang et al. [18]. As well as the evaluation of the key enzyme activities involved in essential oil biosynthesis, including L-phenylalanine aminolyase, 3-deoxy-D-arabino-heptulosonate-7-phosphate synthase (DAHPS) and O-methyltransferase [18].

### 2.4. Seed Preparation for Biological Activity Assay

Seeds from each accession cultivar under each treatment were pulverized separately, and around 4 g powder was extracted in ethanol at room temperature for 24 h. The supernatant was filtered using a Whatman No.1 filter paper after centrifugation at 8000× *g* for 25 min. The samples were kept at −20 °C until testing, after concentrating the extract with a rotary evaporator (IKA-WERKE-RV06ML, Staufen, Germany).

### 2.5. Hypocholesterolemic Activity

#### 2.5.1. Inhibition of Micellar Solubility of Cholesterol

The effect of aniseeds on the micellar solubility of cholesterol was measured according to the method described by Okla et al. [17]. Concentrated seed extract was added to 7 mL of micellar solution (2 mM cholesterol, 10 mM sodium taurocholate, 132 mM NaCl, 5 mM oleic acid, 15 mM sodium phosphate, pH 7.4) at the rate of 10 mg/mL. The mixture was sonicated for 2 min before being incubated for 24 h in a water bath at 37 °C. The micellar solution was then ultracentrifuged at 40,000 rpm for 60 min at 20 °C, and 10 µL of the supernatant was utilized for an enzymatic assay to determine the cholesterol content at 500 nm, using a cholesterol measurement kit (Pointe Scientific, C7510, Fisher Scientific, Hampton, NH, USA). Then, the inhibition activity of the micellar solubility of cholesterol was calculated for each sample as follows:

Inhibition activity (%) = [(C − S)/C] × 100

where C is the cholesterol concentration in the control micellar solution, and S is the cholesterol concentration in a micellar mixture containing seed powder.

#### 2.5.2. Pancreatic α-Amylase Inhibition Assay

The seed extract was combined with a reaction solution of starch (1 g/L) and phosphate buffer (pH 6.9) to measure pancreatic α-amylase inhibition activity. The reaction was started by adding 3 U/mL of amylase enzyme. Then, 500 μL of dinitrosalicylic (DNS) reagent was added after 10 min of incubation to stop the reaction. Afterward, the mixture was boiled at 100 °C for 10 min. Finally, 500 µL of a 40% potassium sodium tartrate solution was added to the mixture. The absorbance level was measured at 540 nm.

#### 2.5.3. Pancreatic Lipase Inhibition Assay

The inhibitory activity of the seed extract against pancreatic lipase was measured by using 4-MUO as a substrate [19]. Briefly, 0.5 mL of different concentrations of seed extract was added to 0.5 mL freshly produced lipase (1 mg/mL; lipase from porcine pancreas, Sigma-Aldrich, St. Louis, MI, USA). The mixtures were centrifuged at 4000 rpm for 10 min, after 10 min of stirring, and 2 mL of 4-MUO (0.1 mM) solutions were added. As a blank, a reaction mixture without seed extract was employed. The mixture was incubated at 37 °C. At different time points, aliquots of 0.2 mL were obtained, and 4-MUO hydrolysis by lipase was detected at 350 nm excitation and 450 nm emission wavelengths. The IC50 values (mg/mL) were calculated using a logarithmic regression curve, which was defined as the concentration of the extract that inhibited 50% of the pancreatic lipase activity.

### 2.6. Antioxidant Capacity

In vitro antioxidant capacity was measured by diphenylpicrylhydrazyl (DPPH) and ferric reducing antioxidant power (FRAP) [20]. About 0.2 g of seed powder was extracted in ethanol at 80% and centrifuged (14,000 rpm, 20 min). By combining 0.1 mL of diluted seed extract with 0.25 mL of DPPH solution or FRAP reagent, the antioxidant capacity was determined. The absorbance was measured at 517 nm and 600 nm, respectively, using the spectrometric method, after incubation at room temperature.

### 2.7. Anti-Lipid Peroxidation

Using egg yolk homogenate as a lipid-rich medium, the degree of lipid peroxidation was calculated using thiobarbituric acid reactive substances (TBARS). First, 15 mM ferrous sulfate was combined with seed extract and 0.5 mL of 10% (*v*/*v*) egg yolk homogenate. Then, 1.5 mL of 10% tri-carboxylic acid (TCA) was added after 30 min of incubation. After incubation, the mixture was transferred to a tube containing 1.5 mL of thiobarbituric acid (TBA) at 0.67% and boiled for 30 min. The chromogen formed was measured at 535 nm.

### 2.8. Statistical Analysis

One-way and three-way analyses of variance (ANOVA) were performed on all of the data (SPSS). For subsequent pairwise statistical comparisons of means, Duncan’s test was employed (SPSS). Additionally, R was used to perform principal component analysis (PCA). Individual sample distributions in the first two PCA dimensions were visualized using PCA graphs. The parameters and the degree to which they contribute to the total variation explained by the first two PCA dimensions were shown as arrows.

## 3. Results and Discussion

### 3.1. Seed Maturation Increasing Dry Biomass, Seed Yield, and Nutrient Accumulation

**Figure 1:**
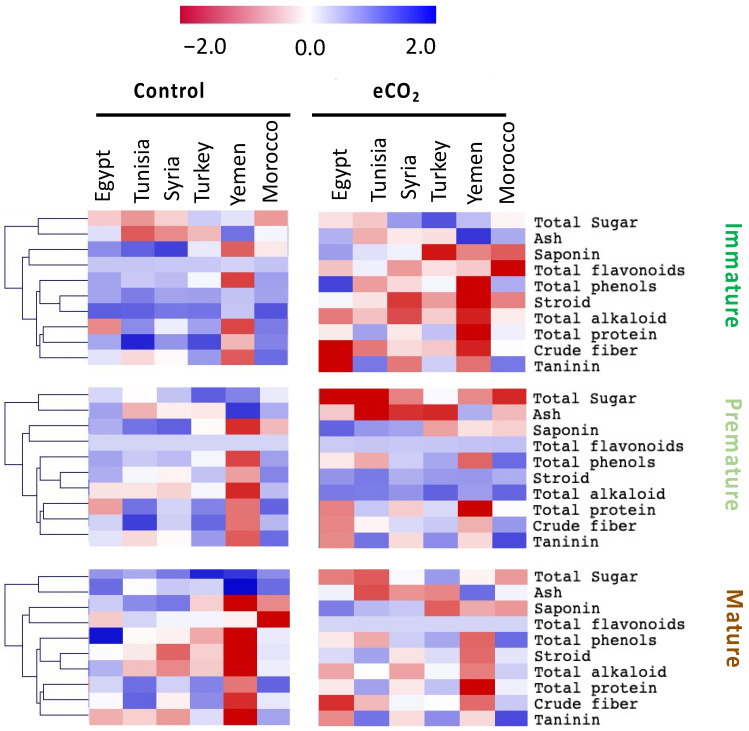
Heatmap showing the hierarchical clustering analysis of total nutrients of six *Pimpinella anisum* L. accessions at three seed developmental stages and under control and elevated CO_2_ growth conditions. The level patterns are relatively demonstrated on the heatmap based on the mean value (*n* = 5) for each parameter. Red and blue color gradients indicate higher and lower levels, respectively.

The variation in seed chemical composition is clearly dependent on the ripening stages [21], where the changeover of the seed from the immature to mature stages is characterized by a gradual change in their dry weight and chemical composition [5]. Obviously, mature seeds from all geographical sources showed significantly higher (*p* < 0.05) values of DW% and seed yield per plant than did premature and immature seeds (Table 1). The highest levels of DW% were recorded in seeds from Egypt and Morocco. Regarding the nutrition level, the improved levels of primary and secondary active metabolites, such as essential oil, essential amino acids, phenolic acid, and flavonoids are involved in improving the food nutritive values [9]. By scavenging free radicals, phenols and flavonoids serve as natural antioxidants [22]. Here, the effect of maturity on the total nutrients was significant but variable from one nutrient to another. Similarly, the chemical composition significantly varied during the seed maturation of *Apiaceae* seeds [10,21]. Overall, the nutritive value of mature anise seeds was significantly greater than in premature and immature ones (Figure 1, Supplementary Table S2), whereas the levels of saponin, steroids, total protein, total alkaloid, crude fibers and tannin, total sugars, total oil, and total phenolics in anise seeds significantly increased at the mature stage. Moreover, we observed significant interactions between accession, maturity stage, and eCO_2_ treatment for most measured parameters (Supplementary Table S1).

Although there are very limited sources in the literature on the chemical composition variation of anise seeds during maturation, we know that the biosynthesis of total nutrients is very active at the mature stage of seed development [5]. For instance, the regulation of oil accumulation during the mature stage is associated with fatty acid synthetase activation [10]. Likewise, the total phenolic acids were increased in fennel fruit as maturity progressed [12]. Moreover, the chemical composition significantly varies during the seed maturation of *Apiaceae* seeds [10,23]. In this regard, the maturation progresses upregulated the expression of the genome that activates the enzyme activities involved in the biosynthesis of bioactive compounds [10].

Thus, given the nutritional importance of measured metabolites, knowledge of their accumulation during seed development maturation is important to identify the right time for seed harvesting.

### 3.2. eCO_2_ Improved the Nutritive Values of Seeds, Particularly at the Mature Stage of Aniseed

Currently, several research projects are trying to find innovative methods to improve plant growth, metabolism, biological activity, and resistance. Among these methods, acidic electrolyzed water, ultraviolet irradiation, laser irradiation [17], and elevated atmospheric CO_2_ (eCO_2_) [20,24,25] are receiving much interest. In the current study, the dry weight percentage of anise seeds from different geographical locations (Egypt, Tunisia, Syria, Turkey, Yemen, and Morocco) harvested from plants grown either with eCO_2_ or aCO_2_ were determined at different maturity stages, i.e., immature, premature and mature (Table 1).

The application of eCO_2_ led to significant elevations in DW% and/or seed yield on most occasions, as compared to aCO_2_, except in seeds; the effectiveness of the high level of CO_2_ was clearer in mature seeds. In this regard, eCO_2_ increased the yields of carrots, radishes, and turnips [26]. The high yield in seeds from anise plants exposed to eCO_2_ could be attributed to an improvement in the photosynthesis process as a result of eCO_2_ exposure. Since CO_2_ is a crucial substrate for the plant photosynthesis process, eCO_2_ thus directly promotes plant photosynthesis by altering the chemical composition of the plant by changing the carbon and nitrogen metabolism, as well as increased carbon gain [26,27]. As a result, it supplies the metabolic energy needed for the biosynthesis of different nutrients and metabolites [27,28]. For instance, eCO_2_ could boost the biosynthesis of antioxidants, including phenols and flavonoids, by increasing the availability of the C-skeleton in combination with a sufficient supply of inorganic matter [28].

Herein, the effect of eCO_2_ on the levels of total nutrients in anise seeds from different locations including Egypt, Tunisia, Syria, Turkey, Yemen, and Morocco at different maturity stages was recorded (Figure 1, Supplementary Table S2). We also measured significant interactions between the three factors (accession, maturity stage, and eCO_2_ treatment) (Supplementary Table S1). Regarding the effectiveness of eCO_2_ in enriching the total nutrient levels in anise seeds, it had negative effects on the levels of most measured nutrients at the immature stage. On the contrary, eCO_2_ showed an improving effect on the levels of total nutrients, i.e., saponin, steroids, total protein, total alkaloid, crude fibers, and tannin, and on total sugars in anise seeds at the mature stage. In the present study, eCO_2_ significantly enriched the levels of total phenols and flavonoids in mature seeds. In line with our findings, [25] reported significant elevations in phenols and flavonoids in eCO_2_-treated seeds. Accordingly, eCO_2_ could be an efficient way to enhance the nutraceutical properties of agricultural foods and herbal plants, including anise seeds. In this regard, eCO_2_ has significantly increased the contents of protein, total lipids, carbohydrates, and fibers in treated alfalfa sprouts [25].

### 3.3. Reduced Essential Oil Content and Metabolism by Maturation, as Mitigated by eCO_2_

**Figure 2:**
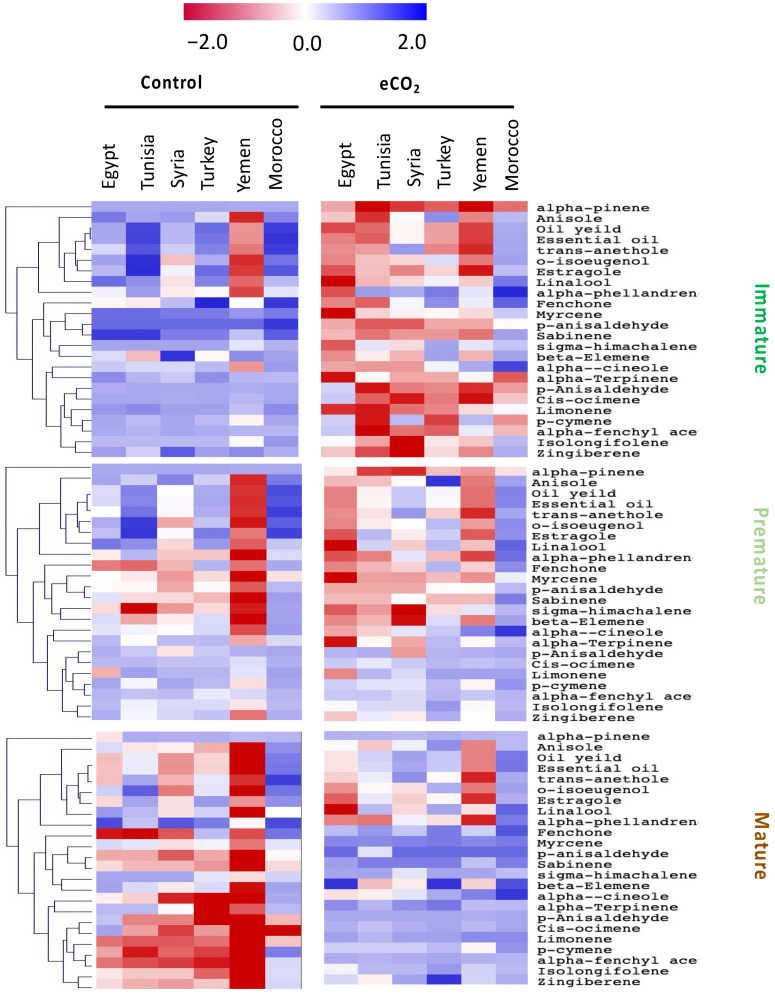
Heatmap showing the hierarchical clustering analysis of essential oils levels of six *Pimpinella anisum* L. accessions at three seed developmental stages, and under control and elevated CO_2_ growth conditions. The level patterns are relatively demonstrated on the heatmap, based on the mean value (*n* = 5) for each parameter. Red and blue color gradients indicate higher and lower levels, respectively.

The quality of anise is mainly determined based on its essential oil content and its composition. In the study by [3], (E)-anethole, eugenyl acetate, g-gurjunene, and estragole represent 90%, 2%, 1.85%, and 1.04% of the total essential oil content, respectively. Another study by [29] reported that anise seeds contain up to 6% of essential oil, consisting primarily of trans-anethole, besides up to 12% of oil rich in fatty acids, especially petroselinic acid [6]. The essential oil content and chemical composition are the most important factors in determining the quality of anise [30]. Both parameters are significantly influenced by environmental factors, such as weather conditions [11]. Herein, to investigate the effect of eCO_2_ on the essential oil content of anise seeds at different maturity stages, we measured 22 individual essential oils, besides the total percentage of essential oil and oil yield percentage in treated and untreated anise seeds at three maturity stages (immature, premature and mature) (Figure 2, Supplementary Table S3). The total essential oil percentage and concentrations of most measured essential oil gradually decreased with maturity in untreated plants from various locations. Similarly, plant seeds at various levels of maturity showed changes in the yield and quality of their essential oils [1]. Regarding the effect of eCO_2_ on essential oil levels, it was clear that it significantly interacted with maturity-stage and accession cultivars (Supplementary Table S1). The effect was negative at the immature stage, as the levels of most essential oils were significantly lower in eCO_2_-treated seeds than control seeds (*p* < 0.05), while the effect of eCO_2_ on essential oil content at the premature stage was negligible or reducing on most occasions. On the other hand, eCO_2_ had an improving effect on essential oil content in anise seeds at the mature stage (*p* < 0.05). To sum up, the effect of eCO_2_ treatment and maturity on oil yield, essential oil yield, and composition in anise seeds, immature seeds from untreated plants were the richest in most parameters, followed by eCO_2_-treated mature seeds, and then premature, untreated seeds. However, immature treated seeds were the poorest in essential oil concentrations (Figure 2, Supplementary Table S3).

The effect of maturity on the essential oil contents of anise (*Pimpinella anisum* L.) seeds [5] and cumin (*Cuminum cyminum* L.) seeds [10] was previously investigated. Similar to this study, [5,11] reported that during anise seed maturity, there were significant changes in oil content (*p* < 0.05). These studies stated that the essential oil yield of aniseed declined with the ripening process, and the maximum level was reported at the immature stage, which can be explained by the fact that the number of fruits in the early stage was higher than in the last one. Additionally, several studies reported a decrease in the total oil yield with maturing, such as in *Olea europeae* [31] and *Rhus tripartitum* [32].

Improved photosynthesis due to high CO_2_ exposure consequently led to boosting the primary metabolism of the plant, represented by elevations in the concentrations of essential oils and their precursors, such as the amino acid phenylalanine and the related biosynthetic enzyme phenylalanine ammonialyase [33].

Essential oils also are mainly composed of phenylpropanoid compounds, whereas cinnamic acid and p-coumaric acid have been important secondary metabolites in essential oil biosynthesis in plants [18]. Phenylpropanoid compounds are biosynthesized from phenylalanine via shikimic and cinnamic acids [34]. The enzyme 3-deoxy-d-arabino-heptulosonate-7-phosphate synthase (DAHPS) catalyzes the first enzymatic step in the pathway of shikimic acid [35]. Shikimic acid is converted to chorismite, which is a precursor for phenylalanine biosynthesis. Consequently, the phenylalanine aminolyase enzyme (PAL) catalyzes the conversion of phenylalanine to cinnamic acid, which is followed by the production of p-coumaric acid from cinnamic acid [36].

Therefore, in this study, the effect of eCO_2_ on the levels of phenylalanine, L-phenylalanine amino-lyase, DAHPS, cinnamic acid, shikimic acid, and O-methyltransferase in anise seeds collected from different locations including Egypt, Tunisia, Syria, Turkey, Yemen, and Morocco at different maturity stages was investigated (Table 2). Mostly, eCO_2_ significantly induced (*p* < 0.05) the accumulation of these metabolites and enhanced the enzymatic activities in anise seeds from various origins, indicating their interaction between CO_2_ treatment and origin (Supplementary Table S1). Immature seeds from most locations had significantly higher values (*p* < 0.05) of phenylalanine, L-phenylalanine aminolyase, DAHPS, and O-methyltransferase; then, these metabolites and enzyme activities decreased with maturity. On the other hand, cinnamic acid and shikimic acid levels were significantly lower in immature seeds, then they significantly increased (*p* < 0.05) with maturity in anise seeds from most locations (Table 2). Out of these metabolites and enzymes, apparently, shikimic acid was the most predominant one; furthermore, eCO_2_ exposure significantly elevated (*p* < 0.05) the shikimic acid level in treated seeds. L-phenylalanine aminolyase came in at the second place of the most predominant ones; eCO_2_ also had a positive effect on this activity in anise seeds. These obtained data could explain the enhancement in essential oil metabolism in mature anise seeds, caused by eCO_2_ exposure. Additionally, they could suggest mature anise seeds treated with eCO_2_ as a promising nutraceutical and pharmaceutical compound.

### 3.4. eCO_2_-Treated Mature Aniseeds Showed the Highest Biological Activity

Anise seeds essential oils are well known for their nutritive and biological value as antimicrobial, anti-inflammatory, antispasmodic, and antioxidant compounds [37]. Pancreatic lipase and amylase inhibition reduce hyperlipidemia, whereas dietary triacylglycerols, the main lipid constituent of the human diet, must be hydrolyzed by pancreatic lipase before they can be consumed [38]. Furthermore, lowering micellar solubilization helps to delay cholesterol absorption in the small intestine [39]. In addition, free-radical scavenging is essential to inhibiting cholesterol oxidation into low-density lipoprotein (LDL) [40]. Plant phenolics, saponins, and alkaloids have been shown to inhibit lipid-metabolizing enzymes and reduce cholesterol’s micellar solubility [38]. Additionally, plant polyphenols and vitamins are well-known for their free-radical scavenging capacity [41]. The hypocholesterolemic potential of anise seeds was previously recorded in vivo by [42], who reported a significant reduction in the serum cholesterol of birds fed on a diet containing anise essential oil.

To assess the hypocholesterolemic activity of eCO_2_-treated and untreated anise seeds from various locations at different maturity stages, we measured the pancreatic amylase and lipase activities and inhibition of cholesterol micellar solubility activity. The highest amylase activity was found in mature seeds. In addition, eCO_2_ significantly reduced (*p* < 0.05) the amylase activity of seed extracts from almost all geographical locations in all maturity stages. Interestingly, the highest amylase activity was recorded in seeds from Yemen (Table 3). A similar scenario was noticeable in the case of lipase activity, as eCO_2_ significantly decreased (*p* < 0.05) the lipase activity at all maturity stages in seeds from different locations. In contrast to amylase activity, the highest levels of lipase activity were mostly found in immature seeds. While similar to amylase activity, anise from Yemen had the highest levels of lipase activity in comparison with other locations (Table 3). As regards the anti-cholesterol activity of seed extracts, in terms of the inhibition of cholesterol micellar solubility, even though the best maturity stage for this activity was immature seeds, eCO_2_ had a negative reducing effect on activity at this stage. On the contrary, the effect of eCO_2_ on this activity in premature and mature seeds was enhancing, as it led to significant increases in the anti-cholesterol ability at these stages (*p* < 0.05). (Supplementary Table S1). Anise from Yemen had the highest inhibition activity of cholesterol micellar solubility as compared to other sources (Table 3). Following the current study, a significant elevation in the in vitro hypocholesterolemic effect of fenugreek seeds as a result of eCO_2_ treatment, as indicated by the inhibition of cholesterol micellar solubility and pancreatic lipase activity, was reported [8]. The researchers attributed that to the eCO_2_-induced improvements in the levels of saponins, phenolics, vitamins, and alkaloids in seeds from treated plants. Purified plant saponins, polyphenols, and alkaloids have been reported to have hypocholesterolemic properties, both in vitro and in vivo [38,43]. Therefore, the enhancement in the hypocholesterolemic potential of aniseeds from eCO_2_-treated plants could be ascribed to the reported eCO_2_-inducing effect of the levels of total phenols, flavonoids, alkaloids, and saponins.

In addition to a bioactive essential oil, the seeds of anise are rich in antioxidants, such as phenolic acids and flavonoids [6]. Free radicals and reactive oxygen species in biological systems and foods have been shown to have adverse effects on living organisms and food quality. Thus, these natural sources of antioxidants have attracted the interest of many researchers during the last few decades.

In this context, the antioxidant activity of anise seeds as represented by DPPH (%) was evaluated at different maturity stages under eCO_2_ and ambient air conditions (Table 3). The most suitable maturity stage for a high DPPH percentage was immature seeds, as this parameter decreased with maturity. Interestingly, anise seeds cultivated at eCO_2_ had significantly higher levels of diphenyl picrylhydrazyl (DPPH) than control seeds (*p* < 0.05). Regarding ferric reducing antioxidant power (FRAP), their values were significantly enhanced (*p* < 0.05) by eCO_2_ in anise samples collected from Egypt and Tunisia, while in anise collected from other locations, the FRAP levels were mostly reduced. Immature seeds showed the highest values of FRAP in comparison with other stages. Regarding anti-lipid peroxidation, the effect of CO_2_ levels was not significant. On the other hand, the effect of seed maturity was clear, as immature seeds had the highest levels of anti-lipid peroxidation, whereas mature seeds had the lowest levels of anti-lipid peroxidation (Table 3). The higher antioxidant capacity in immature anise seeds compared to mature ones, as reported in this study, could be attributed to the reported higher levels of phenolics, flavonoids, and essential oils in immature seeds in comparison with mature ones. The antioxidant activity of anise seeds from four locations (Egypt, Turkey, Tunisia, and Serbia) was previously evaluated at different maturity stages [5] based on DPPH, chelating ability, and reducing power assays. Conversely to our present findings, the authors of [5] reported that maximum antioxidant activity was detectable in aniseed extracts at the mature stage. These variations could be ascribed to the variations among geographical locations, as well as environmental, variety, and genetic factors [11,12]. Similarly, the total antioxidant capacities in terms of FRAP, oxygen radical absorbance capacity (ORAC), and the inhibition of LDL oxidation of *Thymus vulgare* under the effect of eCO_2_ were significantly improved [44]. They attributed such an effect to the boost in their contents of volatile oils, flavonoids, and phenolic acids, such as cinnamic and rosmarinic acids [45].

Overall, the variation in biological activities among different maturity stages and provenances in response to eCO_2_ treatment could be attributed to the aforementioned variations in total nutrients, phenolics, flavonoids, saponin, alkaloids, and essential oil yield and composition under control and eCO_2_ conditions among aniseeds from different locations at various maturity stages, which could be ascribed to environmental and genetic factors [5,11].

### 3.5. Accession and Developmental Stage-Specific Effect on Seed Chemical Composition and Biological Activity of eCO_2_-Treated Aniseeds

The dynamic change in seed chemical composition is dependent on the seed developmental stage and plant cultivars/accession [46]. Moreover, the chemical composition is the most important factor in determining the quality of anise. The parameters are significantly influenced by environmental factors such as soil type and weather conditions, particularly during anise fruit development (plant maturity stages), as well as by the agronomic practices used [11].

**Figure 3:**
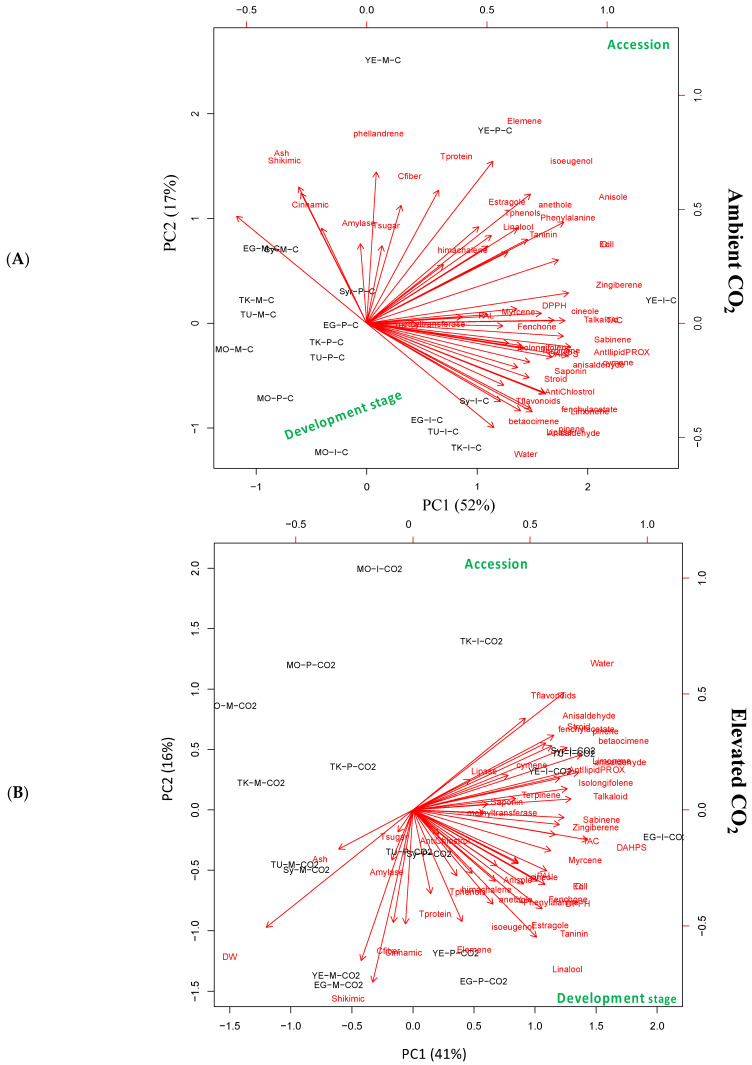
Principal component analysis (PCA) of seed chemical compositions and biological activities of six *Pimpinella anisum* L. accessions at three developmental stages under (**A**) ambient CO_2_ (control) or (**B**) elevated CO_2_ growth conditions.

To better understand the accession-specific responses, we performed a principal component analysis (PCA) of seed chemical compositions and biological activities at the three developmental stages, under severe ambient CO_2_ (control) or eCO_2_ treatments (Figure 3). Under control conditions, we observed a clear separation between the accession’s parameters along the PC1, which explains about 52% of the total variation (Figure 3A). Remarkably, the anise seeds originating from Yemen have been separated from anise seeds originating from other locations. The seeds from untreated anise originating from Yemen were the poorest ones in total and individual essential oil contents (*p* < 0.05). There was also a clear separation between the parameters of the immature stage (I), and mature (M) and premature (P) stages of all accessions along PC2 (representing 17% of the total variation) (Figure 3A). Seed at the immature stages showed low antioxidant, PAL, and anticholesterolemic activities and flavonoids, saponin, steroids levels, but a high content of several essential oils. Under eCO_2_ conditions, the PCA bi-plot also grouped accession and seed developmental stages according to their response to eCO_2_. PC 1 explained 41% and PC2 explained 16% of the total variance (Figure 3B). Similar to the results of PCA analysis of parameters under control conditions, the parameters of anise seeds at the immature stage are markedly separated from other developmental stages across PC1.

The responses of accessions were clearly separated along the PC2, whereas Morocco and Turkey accessions showed specific responses to elevated CO_2_. Together, these data show that the anise accessions at different seed developmental stages were differentially grouped; this also indicated the specificity of nutritive metabolite accumulation in response to CO_2_ treatment. In this regard, several other studies have also indicated the effect of provenance on the chemical composition of aromatic seeds and herbal plants [6,23,47]. For instance, a variation in chemical composition and antioxidant activity of four aniseed populations showed accession-specific responses [5], whereas the observed differences in their antioxidants were closely related to genetic factors [23]. Moreover, the maturity stage can significantly affect seed chemical composition, where, for example, the ripening stage showed fewer increases in essential oil accumulation compared to other stages [11]. Additionally, the essential oil content of anise might significantly differ among anise fruits from different origins [48]. Furthermore, the variation in oil yield and composition among seeds from different locations was noticeable in the current study (Figure 2 and Supplementary Materials Table S2). This could be ascribed to environmental and genetic factors [5,11]. Similarly, Oezel [12] reported that the chemical variability in essential oils in aromatic seeds was ascribed to geographical origin, environmental conditions, and genetic factors.

## 4. Conclusions

To conclude, eCO_2_ demonstrated inducing properties on the levels of nutrients, antioxidant metabolites, essential oil metabolism, and biological activity of aniseeds, yet its effectiveness is related to maturity stages and provenances. eCO_2_-treated aniseed accessions from Tunisia, Syria, Turkey, Morocco, and Egypt showed significant elevations in total nutrients and antioxidant metabolites. Interestingly, the total and individual essential oil levels decreased with seed maturation, while eCO_2_ reversed this effect. Essential oil metabolism-related precursors and key enzyme activities were enhanced by eCO_2_ treatment. Consequently, eCO_2_ promoted the antioxidant and hypocholesterolemic activities of aniseeds, particularly at mature stages. Further studying of eCO_2_-treated aniseeds as a nutraceutical and pharmaceutical product is suggested.

## Figures and Tables

**Table 1:** Dry weight percentage and fruit yield per plant of the six *Pimpinella anisum* L. accessions at three seed developmental stages and under control and elevated CO_2_ (eCO_2_) growth conditions. Data are represented by the means of at least 5 replicates ± standard error. Different small letter superscripts within a row indicate significant differences between control and eCO_2_-treated samples at *p* < 0.05.

Plant Source	Maturity Stage					
	Mature		Premature		Immature	
	Control	eCO_2_	Control	eCO_2_	Control	eCO_2_
Dry weight (%)						
Egypt	82.8 ± 0.97 ^e^	84.8 ± 0.79 ^e^	56.2 ± 1.13 ^c^	63.4 ± 1.18 ^d^	14.1 ± 0.49 ^a^	17.2 ± 0.2 ^b^
Tunisia	67.1 ± 0.65 ^d^	81 ± 2.56 ^e^	47.8 ± 0.03 ^c^	54.2 ± 0.59 ^c^	12.6 ± 0.11 ^a^	14.5 ± 0.07 ^b^
Syria	76.2 ± 0.74 ^d^	69 ± 0.64 ^bc^	54.3 ± 0.03 ^b^	50.5 ± 0.55 ^b^	14.3 ± 0.13 ^a^	13.5 ± 0.06 ^a^
Turkey	67.3 ± 1.4 ^c^	72.7 ± 2.07 ^d^	46.4 ± 0.2 ^b^	49.4 ± 0.2 ^b^	12.3 ± 0.04 ^a^	13.1 ± 0.05 ^a^
Yemen	66 ± 0.62 ^d^	79.3 ± 1.1 ^e^	45.9 ± 4.03 ^c^	44.1 ± 0.37 ^c^	19.9±0.07 ^b^	11.7 ± 0.03 ^a^
Morocco	82.3 ± 1.6 ^e^	80.1 ± 1.6 ^e^	43.5 ± 0.3 ^d^	26.5 ± 0.7 ^c^	11.5 ± 0 ^a^	16.5 ± 0.1 ^b^
Fruit yield per plant (g)						
Egypt	0.12 ± 0.05 ^a^	0.19 ± 0.02 ^b^	0.10 ± 0.02 ^a^	0.14 ± 0.07 ^b^	0.08 ± 0.01 ^a^	0.10 ± 0.04 ^a^
Tunisia	0.17 ± 0.05 ^bc^	0.23 ± 0.06 ^c^	0.12 ± 0.03 ^a^	0.20 ± 0.05 ^c^	0.10 ± 0.03 ^a^	0.14 ± 0.06 ^b^
Syria	0.23 ± 0.04 ^ab^	0.34 ± 0.04 ^c^	0.20 ± 0.01 ^a^	0.31 ± 0.01 ^c^	0.17 ± 0.01 ^a^	0.21 ± 0.01 ^ab^
Turkey	0.18 ± 0.04 ^b^	0.27 ± 0.07 ^d^	0.16 ± 0.04 ^ab^	0.21 ± 0.04 ^c^	0.11 ± 0.04 ^a^	0.18 ± 0.01 ^b^
Yemen	0.15 ± 0.02 ^b^	0.29 ± 0.01 ^d^	0.15 ± 0.02 ^b^	0.20 ± 0.01 ^c^	0.08 ± 0.02 ^a^	0.11 ± 0.02 ^ab^
Morocco	0.13 ± 0.01 ^ab^	0.29 ± 0.06 ^c^	0.10 ± 0.01 ^a^	0.14 ± 0.06 ^b^	0.08 ± 0.01 ^a^	0.12 ± 0.01 ^ab^

**Table 2:** Essential oil-related precursors and related enzyme activities of six *Pimpinella anisum* L. accessions at three seed developmental stages and under control and elevated CO_2_ (eCO_2_) growth conditions. Data are represented by the means of at least 5 replicates ± standard error. Different small letter superscripts within a row indicate significant differences between control and eCO_2_-treated samples at *p* < 0.05.

Parameters (mg/gFW)	Plant Source	Maturity Stages					
		Mature		Premature		Immature	
		Control	eCO_2_	Control	eCO_2_	Control	eCO_2_
Phenylalanine	Egypt	3.3 ± 0.26 ^a^	4.3 ± 0.1 ^b^	3.9 ± 0.14 ^a^	4.9 ± 0.03 ^b^	4.4 ± 0.06 ^b^	5.4 ± 0.02 ^c^
	Tunisia	3 ± 0.1 ^a^	3.6 ± 0.34 ^a^	3.4 ± 0.1 ^a^	4 ± 0.1 ^ab^	3.8 ± 0 ^a^	4.4 ± 0.09 ^b^
	Syria	3.1 ± 0.08 ^a^	3 ± 0.1 ^a^	3.5 ± 0.03 ^a^	3.3 ± 0.05 ^a^	3.9 ± 0.01 ^b^	3.7 ± 0.03 ^ab^
	Turkey	2.8 ± 0.18 ^a^	2.6 ± 0.12 ^a^	3.2 ± 0.12 ^b^	2.8 ± 0.08 ^a^	3.6 ± 0.07 ^b^	3.1 ± 0.04 ^c^
	Yemen	4.9 ± 0.08 ^b^	2 ± 0.08 ^a^	5.5 ± 0.02 ^b^	2.2 ± 0.05 ^a^	6.1 ± 0.01 ^b^	2.5 ± 0.03 ^a^
	Morocco	1.6 ± 0.2 ^a^	1.47 ± 0.1 ^a^	1.8 ± 0.1 ^a^	1.57 ± 0.1 ^a^	2.0 ± 0.1 ^ab^	1.9 ± 0 ^a^
L-phenylalanine aminolyase	Egypt	20.3 ± 0.39 ^a^	32.7 ± 1.3 ^b^	48.9 ± 2.3 ^c^	74.5 ± 4.8 ^d^	51.9 ± 0.97 ^c^	82.3 ± 1.9 ^e^
	Tunisia	19.4 ± 0.5 ^a^	25.9 ± 1.8 ^b^	39.9 ± 2.3 ^b^	47.1 ± 4.4 ^d^	33.3 ± 1.2 ^b^	45.2 ± 5.41 ^c^
	Syria	21.6 ± 0.79 ^a^	31.2 ± 2.2 ^b^	58.6 ± 2.7 ^d^	87.7 ± 11 ^f^	44.9 ± 3 ^c^	62.3 ± 0.19 ^e^
	Turkey	20.6 ± 0.2 ^a^	22.4 ± 1.6 ^a^	62.4 ± 2.9 ^c^	68.1 ± 4 ^c^	36.6 ± 1.9 ^b^	39.6 ± 3.6 ^b^
	Yemen	35.2 ± 0.7 ^a^	31 ± 0.52 ^a^	98.2 ± 6.6 ^d^	103.3 ± 1.5 ^d^	60.8 ± 3.3 ^c^	55.6 ± 1.01 ^c^
	Morocco	25.6 ± 0.2 ^a^	20.5 ± 0.2 ^a^	60.9 ± 1.3 ^b^	71.7 ± 1.2 ^c^	60.8 ± 2.1 ^b^	66.6 ± 0.4 ^c^
* DAHPS	Egypt	0.2 ± 0.01 ^a^	0.5 ± 0.03 ^b^	0.3 ± 0.03 ^a^	1.2 ± 0.06 ^c^	0.5 ± 0.02 ^b^	1.5 ± 0.06 ^c^
	Tunisia	0.2 ± 0.01 ^a^	0.3 ± 0.02 ^ab^	0.4 ± 0 ^b^	0.45 ± 0.02 ^b^	0.61 ± 0 ^c^	0.8 ± 0.05 ^d^
	Syria	0.3 ± 0.01 ^a^	0.3 ± 0.02 ^a^	0.6 ± 0.03 ^b^	0.6 ± 0.03 ^b^	0.9 ± 0.03 ^c^	0.9 ± 0.04 ^c^
	Turkey	0.2±0.01 ^a^	0.2±0.02 ^a^	0.4±0.01 ^b^	0.5±0.02 ^b^	0.7±0.02 ^c^	0.7±0.06 ^c^
	Yemen	0.4 ± 0.01 ^a^	0.4 ± 0.01 ^a^	0.8 ± 0 ^b^	1.2 ± 0.03 ^b^	1.3 ± 0.05 ^v^	0.9 ± 0.02 ^b^
	Morocco	0.3 ± 0 ^a^	0.2 ± 0 ^a^	0.5 ± 0 ^b^	0.6 ± 0 ^b^	0.9 ± 0 ^c^	0.5 ± 0 ^b^
Cinnamic acid	Egypt	3.1 ± 0.52 ^b^	5.8 ± 0.23 ^c^	1.4 ± 0.03 ^a^	3.4 ± 0.14 ^b^	3.2 ± 0.1 ^b^	1.3 ± 0.05 ^a^
	Tunisia	2.9 ± 0.2 ^ab^	3.8 ± 0.5 ^b^	1.6 ± 0 ^a^	2 ± 0.15 ^a^	3.9 ± 0.1 ^b^	5 ± 0.19 ^c^
	Syria	3.6 ± 0.33 ^b^	3.3 ± 0.17 ^b^	2.1 ± 0.08 ^a^	2.3 ± 0.05 ^a^	2.8 ± 0.02 ^a^	4.5 ± 0.09 ^c^
	Turkey	3 ± 0.34 ^d^	3.3 ± 0.4 ^d^	1.5 ± 0.02 ^c^	1.7 ± 0.09 ^c^	0.55 ± 0.01 ^a^	0.74 ± 0.03 ^b^
	Yemen	5.8 ± 0.57 ^c^	3.5 ± 0.3 ^b^	3 ± 0.01 ^b^	4.9 ± 0.13 ^c^	1.2 ± 0 ^a^	1 ± 0.02 ^a^
	Morocco	3.4 ± 0.2 ^d^	2.3 ± 0.2 ^c^	1.7 ± 0.1 ^b^	2.5 ± 0 ^c^	1.9 ± 0.1 ^b^	0.5 ± 0 ^a^
Shikimic acid	Egypt	75.9 ± 3.4 ^c^	96.3 ± 5.72 ^d^	48.7 ± 1 ^a^	56.8 ± 2.74 ^b^	59.7 ± 1.35 ^a^	68.7 ± 0.26 ^c^
	Tunisia	76.1 ± 2.7 ^c^	77.3 ± 6 ^c^	44.8 ± 1.1 ^a^	54.4 ± 5.6 ^b^	57.1 ± 1.7 ^b^	66.7 ± 4.5 ^bc^
	Syria	72.3 ± 2.9 ^b^	74.3 ± 5.3 ^b^	48.2 ± 1.06 ^a^	48.8 ± 1.6 ^a^	44.2 ± 0.36 ^a^	57 ± 1.6 ^ab^
	Turkey	55.5 ± 1.9 ^d^	53.9 ± 1.8 ^d^	38.3 ± 0.8 ^b^	44 ± 1.6 ^c^	20.5 ± 0.2 ^a^	20.4 ± 0.4 ^a^
	Yemen	103.6 ± 6.8 ^d^	72.8 ± 3.16 ^c^	67 ± 2.19 ^c^	77.6 ± 0.92 ^c^	33.3 ± 3.3 ^a^	26.7 ± 4.2 ^a^
	Morocco	59±5 ^c^	38±2.1 ^b^	36.9±1.8 ^b^	41.7±0.7 ^b^	42.6±1.2 ^b^	14.2±0.2 ^a^
O-methyltransferase	Egypt	7 ± 0.32 ^a^	7.7 ± 0.48 ^a^	20.1 ± 0.9 ^c^	22.3 ± 1.37 ^c^	13.4 ± 0.61 ^b^	16.8 ± 0.91 ^b^
	Tunisia	7.1 ± 0.3 ^a^	15.1 ± 0.6 ^b^	20.6 ± 0.9 ^c^	43.6 ± 1.81	13.7 ± 0.6 ^b^	29.1 ± 1.2 ^d^
	Syria	6 ± 0.27 ^a^	12.5 ± 1.54 ^b^	17.4 ± 0.7 ^c^	36 ± 4.4 ^e^	11.6 ± 0.5 ^b^	24 ± 2.9 ^d^
	Turkey	6.3 ± 0.29 ^a^	9.7 ± 0.6 ^b^	18.3 ± 0.8 ^d^	27.9 ± 1.7 ^e^	12.2 ± 0.5 ^c^	18.6 ± 1.1 ^d^
	Yemen	10 ± 0.66 ^a^	15 ± 0.23 ^a^	28.9 ± 1.9 ^b^	37.3 ± 0.6 ^c^	19.3 ± 1.2 ^ab^	28.9 ± 0.4 ^b^
	Morocco	8.7 ± 0.2 ^a^	10.3 ± 0.2 ^b^	25 ± 0.5 ^cd^	29.7 ± 0.5 ^d^	16.7 ± 0.3 ^b^	19.8 ± 0.3 ^c^

* DAHPS: The enzyme 3-deoxy-D-arabino-heptulosonate-7-phosphate synthase.

**Table 3:** Biological activity of six *Pimpinella anisum* L. accessions at three seed developmental stages and under control and elevated CO_2_ (eCO_2_) growth conditions. Data are represented by the means of at least 5 replicates ± standard error. Different small letter superscripts within a row indicate significant differences between control and eCO_2_-treated samples at *p* < 0.05.

Parameters	Plant Source	Seed Maturity Stages					
		Mature		Premature		Immature	
		Control	eCO_2_	Control	eCO_2_	Control	eCO_2_
Amylase activity IC_50_ (mg/mL)	Egypt	23.7 ± 11.1 ^c^	11.1 ± 0.49 ^b^	11.6 ± 4.7 ^b^	7.4 ± 0.4 ^a^	19.4 ± 8.3 ^c^	6.2 ± 0.39 ^a^
	Tunisia	24.5 ± 0.3 ^d^	6.1 ± 0.4 ^b^	20.5 ± 0.1 ^c^	4.2 ± 0.3 ^a^	15.1 ± 0.2 ^c^	5.4 ± 0.18 ^ab^
	Syria	15.9 ± 1.9 ^b^	25.5 ± 0.07 ^c^	8.8 ± 2.07 ^a^	13.1 ± 0.08 ^b^	12.9 ± 3.9 ^ab^	10 ± 0.07 ^a^
	Turkey	20.7 ± 1 ^e^	8.2 ± 1.8 ^c^	11.1 ± 0.4 ^d^	5.9 ± 0.7 ^b^	8.4 ± 0.3 ^c^	4 ± 0.64 ^a^
	Yemen	35.6 ± 0.8 ^c^	23.1 ± 1.5 ^b^	18.7 ± 0.4 ^ab^	13.1 ± 0.5 ^a^	14.3 ± 0.3 ^a^	15.8 ± 2.3 ^a^
	Morocco	5.4 ± 0.2 ^ab^	8.4 ± 0.4 ^b^	4 ± 0.2 ^a^	5.4 ± 0.2 ^ab^	5.9 ± 0.1 ^ab^	8.1 ± 0.2 ^b^
Lipase activity IC_50_ (mg/mL)	Egypt	1.8 ± 0.11 ^b^	0.3 ± 0.29 ^a^	2.4 ± 0.2 ^d^	1.6 ± 0.18 ^c^	16.9 ± 6.5 ^f^	4.2 ± 0.05 ^e^
	Tunisia	2.1 ± 0 ^ab^	1.3 ± 0.4 ^a^	3.1 ± 0.1 ^b^	1.6 ± 0.2 ^a^	6.9 ± 0.9 ^d^	4.4 ± 0.45 ^c^
	Syria	2.3 ± 0.1 ^a^	2.8 ± 0.07 ^a^	2.9 ± 0.08 ^a^	3.4 ± 0.11 ^b^	12 ± 2.97 ^c^	10.5 ± 0.08 ^c^
	Turkey	2.6 ± 0.1 ^a^	2.6 ± 0.2 ^a^	3.3 ± 0.07 ^b^	3.3 ± 0.12 ^b^	9.3 ± 0.36 ^d^	4.8 ± 0.68 ^c^
	Yemen	4 ± 0.34 ^a^	4 ± 0.34 ^a^	5.2 ± 0.17 ^ab^	4.6 ± 0.24 ^a^	15.9 ± 0.33 ^b^	15.5 ± 1.9 ^b^
	Morocco	2 ± 0.2 ^a^	2.5 ± 0.2 ^a^	2.4 ± 0.1 ^a^	2.6 ± 0.1 ^a^	4 ± 0.2 ^b^	8.3 ± 0.1 ^c^
Anti-Cholesterol (Inhibition of cholesterol micellar solubility) %	Egypt	48 ± 2.11 ^a^	53.1 ± 2.4 ^b^	41.6 ± 1.4 ^a^	47 ± 1.99 ^b^	103 ± 34 ^d^	62.5 ± 2.6 ^c^
	Tunisia	47.9 ± 2.1 ^a^	88.8 ± 2.6 ^c^	42 ± 1.5 ^a^	69.7 ± 1.75 ^b^	118.1 ± 1.9 ^c^	60.2 ± 1.58 ^b^
	Syria	42.8 ± 1.3 ^a^	74.3 ± 7.6 ^c^	38.3 ± 1.03 ^a^	58 ± 4.6 ^b^	74.2 ± 16 ^d^	97.2 ± 2 ^e^
	Turkey	41.7 ± 1.2 ^b^	59.9 ± 3 ^d^	36.5 ± 0.8 ^a^	47.7 ± 6.1 ^b^	74.1 ± 2.7 ^e^	50.3 ± 4.8 ^c^
	Yemen	67.3 ± 3.2 ^ab^	91.2 ± 1.09 ^b^	58.1 ± 2.33 ^a^	70.2 ± 0.7 ^ab^	124.8 ± 3.7 ^c^	109 ± 6.8 ^b^
	Morocco	52.6 ± 0.8 ^ab^	59.9 ± 0.8 ^b^	41.5 ± 0.5 ^a^	44.1 ± 0.6 ^a^	47.8 ± 0.9 ^a^	55.4 ± 1.3 ^ab^
DPPH (%)	Egypt	49.2 ± 3.3 ^a^	58.7 ± 2.6 ^b^	71.6 ± 4.4 ^c^	79.4 ± 4.9 ^d^	84.4 ± 4.9 ^e^	96.7 ± 6.02 ^f^
	Tunisia	55.1 ± 3.4 ^b^	45 ± 5.09 ^a^	78.4 ± 4.4 ^c^	72.1 ± 7.01 ^c^	86.9 ± 4.8 ^d^	79.5 ± 9.12 ^c^
	Syria	46.5 ± 2 ^a^	51.5 ± 3 ^a^	70.7 ± 4 ^b^	79.5 ± 7 ^c^	78.1 ± 4 ^c^	61.4 ± 5 ^b^
	Turkey	29.6 ± 1.3 ^a^	37.7 ± 3.6 ^b^	51.4 ± 3.3 ^d^	49.4 ± 2.5 ^d^	51.1 ± 3.5 ^d^	44.3 ± 3.5 ^cd^
	Yemen	61.1 ± 2.51 ^a^	51 ± 3.1 ^a^	88.8 ± 6.9 ^c^	67.1 ± 3.9 ^ab^	86.5 ± 9.1 ^c^	58.5 ± 3.4 ^a^
	Morocco	30.9 ± 2.1 ^a^	32.4 ± 2 ^a^	48.2 ± 4.4 ^c^	42.2 ± 2.9 ^b^	41.5 ± 3.7 ^b^	38.2 ± 2.6 ^ab^
FRAP (nmol/g FW)	Egypt	30.1 ± 4.65 ^a^	42.6 ± 3.2 ^b^	35.7 ± 4.75 ^a^	51.9 ± 3.1 ^c^	48.5 ± 4.1 ^b^	70.3 ± 5.0 ^d^
	Tunisia	31.2 ± 3.8 ^a^	36.9 ± 5.9 ^a^	37.3 ± 3.8 ^a^	43.8 ± 4.7 ^ab^	53.3 ± 3.5 ^b^	61.9 ± 4.4 ^c^
	Syria	33 ± 3.61 ^a^	36.9 ± 4.7 ^ab^	40.2 ± 3.3 ^b^	42.2 ± 5.0 ^b^	68.7 ± 10.0 ^c^	87.5 ± 5.0 ^d^
	Turkey	29.9 ± 4.52 ^a^	25.2 ± 1.4 ^a^	35.8 ± 3.8 ^b^	31.5 ± 1.5 ^a^	86.2 ± 20.6 ^d^	43.7 ± 2.4 ^c^
	Yemen	53.6 ± 6.1 ^bc^	30.4 ± 2.8 ^a^	66.7 ± 6.9 ^c^	34 ± 2.9 ^a^	98.5 ± 3.47 ^d^	45.3 ± 3.3 ^b^
	Morocco	26.5 ± 3.2 ^b^	20.1 ± 2.1 ^a^	28.9 ± 3.3 ^b^	22.3 ± 2.2 ^a^	33.1 ± 3.4 ^b^	23.5 ± 2.1 ^a^
Anti-lipid peroxidation (TBARS)	Egypt	5.9 ± 0.6 ^a^	7.8 ± 0.6 ^b^	6.7 ± 0.78 ^ab^	9.7 ± 0.6 ^c^	18.9 ± 2.0 ^d^	28.6 ± 2.5 ^e^
	Tunisia	6.7 ± 0.7 ^a^	7.3 ± 0.58 ^a^	7.6 ± 0.8 ^a^	9.4 ± 0.67 ^b^	20.9 ± 1.7 ^c^	28.4 ± 4.1 ^d^
	Syria	6.4 ± 0.27 ^a^	10.9 ± 1.4 ^b^	7.6 ± 0.34 ^ab^	14.2 ± 1.8 ^c^	22.3 ± 1.9 ^d^	22.0 ± 1.7 ^d^
	Turkey	6.1 ± 0.5 ^a^	5.2 ± 0.2 ^a^	11.2 ± 2.2 ^b^	6.8 ± 0.2 ^a^	24.7 ± 0.5 ^d^	18.9 ± 2.5 ^c^
	Yemen	13.3 ± 0.6 ^b^	8.8 ± 0.6 ^a^	17.3 ± 1.1 ^c^	11.2 ± 0.8 ^ab^	38.6 ± 2.7 ^d^	12.3 ± 0.9 ^b^
	Morocco	5.1 ± 0.4 ^a^	4.3 ± 0.3 ^a^	6.4 ± 0.5 ^b^	5.4 ± 0.4 ^a^	7.8 ± 0.5 ^c^	14.9 ± 0.9 ^d^

## Data Availability

The data in this study are readily available upon reasonable request to the corresponding author.

## Supplementary Materials

The following are available online at https://www.mdpi.com/article/10.3390/biology10100979/s1, Table S1: *p* values from thee-way analysis of variance for all measured parameters in levels of six *Pimpinella animus* L. accessions at three seed developmental stages and under control and elevated CO_2_ growth conditions. Table S2: Total nutrients of six *Pimpinella animus* L. accessions at three seed developmental stages and under control and elevated CO_2_ growth conditions. The level patterns are relatively demonstrated on the heatmap based on the mean value ± standard error (*n* = 5) for each parameter. Different small letters within a row indicate significant differences between control and eCO_2_-treated samples at *p* < 0.05. Table S3: Essential oil levels of six *Pimpinella animus* L. accessions at three seed developmental stages and under control and elevated CO_2_ growth conditions. The level patterns are relatively demonstrated on the heatmap based on the mean value ± standard error (*n* = 5) for each parameter. Different small letters within a row indicate significant differences between control and eCO_2_-treated samples at *p* < 0.05.
