# Supplementary material for: Effect of Elevated CO2 on Seed Yield, Essential Oil Metabolism, Nutritive Value, and Biological Activity of Pimpinella anisum L. Accessions at Different Seed Maturity Stages
Source: Biology (Basel). 2021 Sep 29;10(10):979. doi: 10.3390/biology10100979 (PMC8533523; doi:10.3390/biology10100979)
Supplement: Supplementary file 1 [file biology-10-00979-s001.zip › biology-1333080-SI.pdf]

Supplementary Materials

Effect of Elevated CO2 on Seed Yield, Essential Oil Metabolism, Nutritive Value, and Biological Activity of *Pimpinella Anisum* L. Accessions at Different Seed Maturity Stages

Mansour A. Balkhyour 1,*,‡, Abdelrahim H.A. Hassan 2,*,‡, Riyadh F. Halawani 1, Ahmed Saleh Summan 1,3 and Hamada AbdElgawad 4

1 Department of Environmental Science, Faculty of Meteorology, Environment and Arid Land Agriculture, King Abdulaziz University, Jeddah, Saudi Arabia.
2 Department of Food Safety and Technology, Faculty of Veterinary Medicine, Beni-Suef University, Beni-Suef 62511, Egypt.
3 Centre of Excellence in Environmental Studies, King Abdulaziz University, Jeddah, Saudi Arabia.
4 Department of Botany and Microbiology, Faculty of Science, Beni-Suef University, Beni-Suef 62511, Egypt.
* Correspondences: Balkhyour, M. A.: mbalkhyour@kau.edu.sa; Hassan, A.H.A.: abdelrahim@vet.bsu.edu.eg
‡ These authors contributed equally to this study.

Supplementary Table S1. *P* values from thee-way analysis of variance for all measured parameters in levels of six *Pimpinella animus* L. accessions at three seed developmental stages and under control and elevated CO_2 growth conditions.

Paramaters	Accession	Treatment	Maturity	Accession X Treatment	Accession X Maturity	Treatment X Maturity	Accession X Treatment X Maturity
**DW %**	<0.00	0.069	<0.00	<0.00	<0.00	0.004	<0.00
**Seed Yield**	<0.00	<0.00	0.001	<0.00	0.01	0.04	0.01
**Saponin**	<0.00	<0.00	<0.00	<0.00	<0.00	<0.00	<0.00
**Steroid**	<0.00	0.147	<0.00	<0.00	<0.00	0.344	0.05
**Total protein**	<0.00	<0.00	0.128	0.091	0.995	0.827	0.997
**Total Sugar**	<0.00	<0.00	0.02	<0.00	0.845	0.538	0.99
**Ash**	<0.00	0.009	<0.00	<0.00	<0.00	0.002	<0.00
**Crude fiber**	<0.00	<0.00	<0.00	<0.00	0.675	0.103	0.872
**Total phenols**	<0.00	0.208	0.113	<0.00	<0.00	0.994	0.999
**Total flavonoids**	<0.00	0.028	<0.00	<0.00	<0.00	0.006	<0.00
**Total alkaloid**	0.004	0.666	<0.00	0.644	0.923	0.921	0.999
**Tannin**	<0.00	0.075	<0.00	<0.00	0.706	0.992	1
**Oil yield**	<0.00	<0.00	<0.00	<0.00	0.013	0.433	0.129
**Essential oil**	<0.00	<0.00	<0.00	<0.00	0.013	0.433	0.129
**trans-anethole**	<0.00	<0.00	<0.00	<0.00	0.195	0.717	0.776
**o-isoeugenol**	<0.00	0.008	0.088	<0.00	1	0.826	1
**p-anisaldehyde**	<0.00	0.218	<0.00	<0.00	0.025	0.781	0.002
**Anisole**	<0.00	0.273	<0.00	<0.00	0.882	0.605	0.917
**p-Anisaldehyde**	<0.00	0.326	<0.00	<0.00	<0.00	0.544	<0.00
**Estragole**	<0.00	<0.00	0.003	0.027	0.814	0.639	0.997
**α-pinene**	<0.00	0.214	<0.00	<0.00	<0.00	0.13	<0.00
**Limonene**	<0.00	0.054	<0.00	<0.00	<0.00	0.054	<0.00
**Myrcene**	<0.00	<0.00	<0.00	<0.00	<0.00	0.167	<0.00
**Linalool**	<0.00	<0.00	0.001	<0.00	0.937	0.974	0.893
**Cis-β-ocimene**	<0.00	0.148	<0.00	<0.00	<0.00	0.78	<0.00
**Sabinene**	<0.00	0.851	<0.00	<0.00	0.139	0.307	0.439
**p-cymene**	<0.00	0.03	<0.00	<0.00	<0.00	<0.00	<0.00
**Aα-phellandrene**	<0.00	0.027	<0.00	<0.00	0.026	0.375	0.001
**Fenchone**	<0.00	0.001	<0.00	0.099	0.028	0.137	0.696
**1,8-cineole**	<0.00	0.009	<0.00	<0.00	0.579	0.292	0.733
**α-fenchyl acetate**	0.216	0.69	<0.00	0.032	0.241	0.846	0.048

**α-Terpinene**	0.338	0.484	<0.00	0.028	0.337	0.758	0.396
**γ-himachalane**	<0.00	0.293	<0.00	<0.00	<0.00	0.442	<0.00
**Isolongifolene**	<0.00	0.223	<0.00	<0.00	<0.00	0.226	<0.00
**β-Elemene**	<0.00	0.583	0.052	<0.00	0.738	0.926	0.93
**Zingiberene**	<0.00	0.418	<0.00	<0.00	0.439	0.983	0.684
**Phenylalanine**	<0.00	<0.00	<0.00	<0.00	0.832	0.48	0.99
**L-phenyla-aminolyase**	<0.00	<0.00	<0.00	<0.00	<0.00	<0.00	0.011
**DAHPS**	<0.00	<0.00	<0.00	<0.00	<0.00	<0.00	<0.00
**Cinnamic acid**	<0.00	0.001	<0.00	<0.00	<0.00	0.021	<0.00
**Shikimic acid**	<0.00	0.64	<0.00	<0.00	<0.00	0.011	<0.00
**O -methyltransferase**	<0.00	<0.00	<0.00	<0.00	0.184	<0.00	0.665
**Amylase**	<0.00	<0.00	<0.00	<0.00	0.625	0.281	0.574
**Lipase**	0.001	0.006	<0.00	0.002	0.001	<0.00	<0.00
**Anti-Cholesterol**	<0.00	0.494	<0.00	0.094	0.099	<0.00	0.002
**DPPH**	<0.00	0.055	<0.00	0.217	0.474	0.424	0.942
**TAC**	<0.00	0.132	<0.00	<0.00	0.298	0.598	0.622
**Ant-lipid peroxidation**	<0.00	0.63	<0.00	<0.00	0.085	0.717	0.001

Supplementary Table S2. Total nutrients of six *Pimpinella animus* L. accessions at three seed developmental stages and under control and elevated CO_2 growth conditions. The level patterns are relatively demonstrated on the heatmap based on the mean value ± standard error (*n*=5) for each parameter. Different small letter superscripts (a, b, c) within a row indicate significant differences between control and eCO_2-treated samples at $p < 0.05$.

Plant source	Mature seeds		Premature seeds		Immature seeds	
	Control	eCO_2	Control	eCO_2	Control	eCO_2
**Saponin**						
Egypt	28.6 ± 1.9^{ab}	24.2 ± 1^{a}	32.3 ± 1^{b}	27.2 ± 1^{a}	35.6 ± 0.9^{c}	30.5 ± 1^{b}
Tunisia	23.8 ± 0.8^{a}	29.8 ± 1.1^{b}	25.9 ± 0.7^{a}	33.6 ± 1.3^{c}	28.5 ± 0.3^{b}	37.8 ± 0.7^{c}
Syria	20.5 ± 2.7^{a}	31.2 ± 1.4^{b}	23.9 ± 2^{a}	35.1 ± 1.6^{b}	26.5 ± 1^{a}	39.6 ± 0.9^{b}
Turkey	38.7 ± 2^{a}	51.5 ± 3.06^{b}	41.8 ± 1^{a}	58.6 ± 3.5^{bc}	46.3 ± 0^{ab}	66.5 ± 1.8^{c}
Yemen	58.6 ± 2^{c}	44.5 ± 7^{a}	64.1 ± 1^{cd}	50.5 ± 6^{ab}	71.1 ± 0^{d}	54.6 ± 4^{b}
Morocco	43.7 ± 1^{a}	46.5 ± 1.4^{a}	48.9 ± 0.6^{a}	52.5 ± 1.3^{ab}	54.2 ± 0.4^{ab}	58.8 ± 0.6^{b}
**Steroid**						
Egypt	159.4 ± 13^{b}	140.6 ± 6^{a}	169.6 ± 38^{b}	221.3 ± 34^{c}	260.3 ± 80^{d}	253.2 ± 19^{d}
Tunisia	116.5 ± 3^{a}	111.4 ± 12^{a}	254.6 ± 14^{c}	158.2 ± 16^{b}	291.3 ± 16^{d}	297.9 ± 28^{d}
Syria	157 ± 16^{a}	172.1 ± 7^{b}	278 ± 28^{c}	296 ± 26^{c}	442 ± 55^{d}	493.1 ± 40.5^{e}
Turkey	153.8 ± 2^{a}	147.5 ± 8.8^{a}	195.6 ± 13^{b}	224.2 ± 46.1^{b}	320.2 ± 2^{c}	379.1 ± 66^{d}
Yemen	194 ± 6^{b}	146 ± 13^{a}	374.8 ± 20^{c}	436.1 ± 81^{d}	626.1 ± 32^{e}	751.5±13f
Morocco	157 ± 1.7^{b}	171.4 ± 9.3^{c}	124.4 ± 11.3^{a}	233.6 ± 34.1^{d}	234.4 ± 17.7^{d}	410.8 ± 53.9^{e}
**Total protein**						
Egypt	**242.8 ± 41^{c}**	**245.4 ± 22^{c}**	**231.4 ± 43^{bc}**	**196.2 ± 27^{b}**	**164.5 ± 21^{a}**	**195.7 ± 15^{b}**
Tunisia	129.2 ± 10^{a}	158.2 ± 14^{c}	116.2 ± 9.4^{a}	140 ± 12.5^{b}	115.8 ± 5.5^{a}	139.6 ± 7.2^{b}
Syria	176 ± 29^{a}	210 ± 18^{b}	164 ± 29^{a}	197 ± 18^{b}	163 ± 16^{a}	197 ± 10.4^{b}
Turkey	139.5 ± 7^{b}	166.9 ± 19^{c}	124.8 ± 8^{a}	155.3 ± 20^{c}	124.4 ± 4^{a}	155 ± 11^{c}
Yemen	274 ± 12^{b}	336.8 ± 51^{c}	251.9 ± 13^{a}	316.1 ± 52^{c}	251.2 ± 8^{a}	316 ± 30^{c}
Morocco	121.5 ± 7^{b}	187 ± 19.8^{c}	110.2 ± 6.7^{a}	177.6 ± 20.3^{c}	109.9 ± 3.9^{a}	177.5 ± 11.7^{c}
**Total Sugar**						
Egypt	**379.9 ± 46^{c}**	**481.9 ± 11^{e}**	**333.8 ± 23^{b}**	**416 ± 5^{d}**	**302.7 ± 3^{a}**	**368.7 ± 6^{c}**
Tunisia	404.4 ± 47^{b}	494.2 ± 45^{c}	354.9 ± 17^{ab}	432.2 ± 17^{b}	312.3 ± 10^{a}	381 ± 10.3^{b}
Syria	377.3 ± 16^{bc}	414.2 ± 43^{c}	325.2 ± 4^{b}	350.5 ± 22^{b}	289.6 ± 3^{a}	305.7 ± 9.4^{b}
Turkey	329.1±51	350.7 ± 10^{c}	276.9 ± 13^{ab}	305.3 ± 4^{b}	247 ± 1^{a}	270.7 ± 4^{ab}
Yemen	339.6 ± 36^{c}	410.9 ± 14^{d}	294.2 ± 17^{b}	360.4 ± 7^{d}	257.7 ± 8^{a}	322.7 ± 2^{c}
Morocco	402.3 ± 43^{c}	457 ± 18^{d}	343.6 ± 22.6^{b}	402.3 ± 5^{c}	299.7 ± 10^{a}	358.9 ± 5^{b}
**Ash**						
Egypt	147.7 ± 18.4^{c}	172.3 ± 12^{d}	130.4 ± 9.33^{a}	153.2 ± 5.7^{c}	115.9 ± 2.5^{a}	134.9 ± 1.2^{b}
Tunisia	202.6 ± 7.6^{bc}	235.7 ± 3.5^{c}	178.3 ± 2.7^{b}	205.9 ± 0.9^{bc}	155.8 ± 0.4^{a}	179.2 ± 0.1^{b}

**Syria**	188.9±16^{b}	214±5.8^{c}	161.9±4^{ab}	186.9±3^{b}	141.4±1^{a}	163.4±0.8^{ab}
**Turkey**	176.2±40^{bc}	216.2±13^{d}	163.5±10^{b}	190.4±3^{c}	144±2^{a}	165.3±1.03^{b}
**Yemen**	117.1±2^{b}	134.3±1^{c}	101.6±1^{ab}	116.4±1^{b}	88.1±0^{a}	101±0^{ab}
**Morocco**	153.9±0.9^{c}	176.5±0.4^{d}	133.6±0.3^{b}	153.4±0.1^{c}	116±0.1^{a}	133.4±0^{b}
**Crude fiber**						
**Egypt**	12.4±1.6^{a}	18.5±1^{b}	13.7±0.97^{a}	21±1	14.8±0.34^{a}	23.6±0^{b}
**Tunisia**	8.6±0.6^{a}	15.3±0^{b}	9.4±0.2^{a}	17±0.3^{b}	10.5±0.1^{a}	18.9±0.4^{b}
**Syria**	11.9±1.2^{a}	13.9±2^{ab}	13.6±0.75^{ab}	14.7±0.8^{b}	15.4±0.2^{b}	16.1±0.2^{b}
**Turkey**	9.8±1^{a}	13.4±0.5^{b}1	10.6±0^{a}	14.9±0.2^{b}	11.7±0^{a}	16.6±0.09^{c}
**Yemen**	17.1±0.2^{a}	17.3±0.5^{a}	19±0.1^{ab}	19.3±0.4^{ab}	21.1±0^{b}	21.4±0.9^{b}
**Morocco**	11.4±0.5^{a}	12±0.2^{a}	12.9±0.2^{a}	13.4±0.1^{ab}	14.3±0^{b}	14.9±0^{b}
**Total phenols**						
**Egypt**	11.7±1.1^{c}	16.1±1.3^{d}	11.7±1.9^{c}	16.1±2.2^{d}	6.1±0^{a}	8±1.1^{b}
**Tunisia**	13.1±0.8^{a}	18.4±1.1^{b}	13.1±1.5^{a}	18.4±1.9^{b}	15.4±1.7^{a}	18.9±2^{b}
**Syria**	12.6±1^{a}	13.3±0.9^{b}	12.6±2^{a}	13.3±1.6^{ab}	15.8±2^{b}	16.7±2.07^{b}
**Turkey**	14.8±1^{ab}	11.8±0.7^{a}	14.8±1^{ab}	11.8±1.2^{a}	18.5±1^{c}	14.8±1.6^{ab}
**Yemen**	22.4±1^{a}	21±1^{a}	22.3±1^{a}	21±2^{a}	28±1^{b}	26.3±2^{ab}
**Morocco**	11.5±0.^{a}4	9.5±0.3^{a}	11.5±0.7^{a}	9.5±0.6^{a}	14.4±0.9^{b}	12±0.7^{a}
**Total flavonoids**						
**Egypt**	0.5±0.1^{a}	0.7±0.2^{b}	1.1±0^{c}	1.1±0 ^{c}	4.8±1.28^{d}	5.1±0^{d}
**Tunisia**	0.5±0.1^{a}	0.5±0.1^{a}	1.1±0^{b}	1.1±0^{b}	1.1±0 ^{b}	2.1±0.5^{c}
**Syria**	0.6±0.1^{a}	0.6±0.1^{a}	1.1±0^{a}	1.1±0^{a}	2.3±3.45^{b}	6.6±3.06^{c}
**Turkey**	0.6±0^{a}	0.5±0.03^{a}	1.1±0^{b}	1.1±0 ^{b}	2.3±2^{d}	3.9±2^{c}
**Yemen**	1±0.1^{b}	0.4±0.02^{a}	1.1±0^{b}	1.1±0^{b}	3.3±2^{b}	4.8±3^{b}
**Morocco**	0.4±0 ^{a}	0.3±0^{a}	1.1±0^{b}	1.1±0^{b}	23.2±1.3^{c}	19.6±1.1^{c}
**Total alkaloid**						
**Egypt**	38.2±2.2^{a}	50.2±2.9^{b}	119.4±8.8^{d}	146.7±15^{e}	74.9±12^{c}	165.5±13f
**Tunisia**	39±2.2^{a}	51.2±2.9^{b}	119.4±38^{c}	107.3±25^{c}	135.9±19^{d}	134.5±31^{d}
**Syria**	49.4±3^{a}	61.1±4.5^{b}	127±40^{c}	147.6±51.3^{cd}	159.3±50 ^{d}	185.1±64^{e}
**Turkey**	46.7±2^{b}	39.5±2.6^{a}	104.2±28^{c}	103.9±37^{c}	130.6±35^{d}	130.3±47.04^{d}
**Yemen**	82.9±5^{b}	65.2±2^{a}	200.4±59^{d}	163.2±12^{c}	251.4±13^{e}	204.6±65^{d}
**Morocco**	33.6±1.1^{a}	41.2±0.7^{a}	79.3±22.4^{b}	85.8±23^{b}	99.5±8.1^{c}	117±29^{bc}
**Tannin**						
**Egypt**	75.5±2.7^{a}	107.7±4.6^{b}	75.4±4^{a}	107.6±7.9^{b}	99.8±16^{b}	146.6±45^{c}
**Tunisia**	90.4±4^{b}	50.1±5.5^{a}	90.3±7.7^{b}	50±9.5^{a}	92.6±7.9^{b}	51.3±10^{a}
**Syria**	83.3±3^{a}	90.1±4^{ab}	83.2±5.6^{a}	90±7.1^{ab}	104.3±7^{ab}	112±8.9^{b}
**Turkey**	59±2^{a}	56±5.8^{a}	59±4^{a}	55.9±10^{a}	74±5^{c}	70.2±12^{d}
**Yemen**	118.2±2^{ab}	90.5±5^{a}	118.1±4^{ab}	90.4±8^{a}	148.1±25^{c}	113.4±11^{ab}
**Morocco**	48.7±2^{a}	40.9±0.9^{a}	48.6±3.5^{a}	40.9±1.5^{a}	61±4.3^{b}	51.3±1.9^{ab}

Supplementary Table S3. Essential oil levels of six *Pimpinella animus* L. accessions at three seed developmental stages and under control and elevated CO_2 growth conditions. The level patterns are relatively demonstrated on the heatmap based on the mean value ± standard error (n=5) for each parameter. Different small letters superscripts (a, b, c) within a row indicate significant differences between control and eCO_2-treated samples at $p < 0.05$.

Essential oils	Mature		Premature		Immature	
	Control	eCO_2	Control	eCO_2	Control	eCO_2
**Egypt**						
**Oil yield %**	$8.7±0.46^{a}$	$10.9±0.09^{b}$	$9.7±0.35^{a}$	$12.8±0.25^{bc}$	$11.6±0.3^{b}$	$13.8±0.2^{c}$
**Essential oil %**	$2.8±0.15^{a}$	$3.5±0.03^{b}$	$3.1±0.11^{a}$	$4.1±0.08^{c}$	$3.7±0.1^{b}$	$4.1±0.08^{c}$
**Phenylpropanoids**						
trans-anethole	$60.9±3^{a}$	$70.9±1^{b}$	$65.5±2.3^{a}$	$78.1±2.7^{c}$	$72.6±2.35^{b}$	$78.1±2.74^{c}$
o-isoeugenol	$3.8±0.3^{a}$	$5.6±0.33^{c}$	$3.9±0.21^{a}$	$5.7±0.24^{c}$	$4.2±0.22^{b}$	$5.7±0.24^{c}$
p-anisaldehyde	$0.1±0.01^{a}$	$0.1±0.01^{a}$	$0.3±0.01^{b}$	$0.4±0.03^{c}$	$0.4±0.01^{c}$	$0.4±0.03^{c}$
Anisole	$2.6±0.19^{a}$	$3.8±0.24^{b}$	$3.001±0.1^{b}$	$4.5±0.2^{c}$	$3.5±0.08^{b}$	$4.4±0.36^{c}$
p-Anisaldehyde	$0.3±0.08^{b}$	$0.2±0.1^{a}$	$0.291±0.07^{b}$	$0.4±0.47^{c}$	$0.63±0.16^{d}$	$0.85±0.47^{e}$
Estragole	$10.1±0.8^{a}$	$14.5±0.7^{b}$	$10.2±0.6^{a}$	$14.7±0.51^{b}$	$12.2±0.8^{ab}$	$14.7±0.5^{b}$
**Monoterpene hydrocarbons**						
α-pinene	$0.037±0.01^{a}$	$0.034±0.01^{a}$	$1±0^{b}$	$0.12±0^{b}$	$0.97±0.21^{c}$	$1.52±0^{d}$
Limonene	$0.26±0.03^{a}$	$0.3±0.04^{a}$	$1.1±0.2^{b}$	$1.3±0.4^{b}$	$1.4±0.5^{b}$	$1.7±0.3^{b}$
Myrcene	$0.2±0.02^{a}$	$0.6±0.04^{b}$	$2.3±0.12^{c}$	$6.9±0.22^{d}$	$1.7±0.6^{c}$	$6.9±0.22^{d}$
Linalool	$1.9±0.17^{a}$	$5.7±0.32^{b}$	$2±0.16^{a}$	$5.7±0.38^{b}$	$2.3±0.08^{a}$	$5.7±0.4^{b}$
Cis-β-ocimene	$0.3±0.08^{a}$	$0.4±0.12^{ab}$	$0.5±0.05^{b}$	$0.7±0.15^{c}$	$0.27±0.1^{a}$	$0.7±0.15^{c}$
Sabinene	$0.2±0^{a}$	$0.5±0.08^{b}$	$0.7±0.09^{c}$	$1±0.1^{c}$	$0.9±0.1^{c}$	$1±0.1^{c}$
p-cymene	$0.5±0.03^{a}$	$0.7±0.08^{b}$	$0.5±0.02^{a}$	$0.7±0.07^{b}$	$1.4±0.12^{c}$	$0.7±0.07^{b}$
Aα-phellandrene	$2.7±0.18^{b}$	$3.9±0.2^{c}$	$3±0.11^{b}$	$4.3±0.16^{c}$	$1.3±0.06^{a}$	$4.3±0.16^{c}$
**Oxygenated monoterpenes**						
Fenchone	$4.7±0.5^{b}$	$3.9±0.07^{a}$	$6±0.2^{d}$	$5.9±0.05^{c}$	$7.1±0.2^{e}$	$5.9±0.05^{c}$
1,8-cineole	$1.8±0.26^{a}$	$2.4±0.12^{ab}$	$2±0.21^{a}$	$2.8±0.08^{b}$	$2.3±0.12^{ab}$	$2.8±0.08^{b}$
α-fenchyl acetate	$0.049±0.001^{a}$	$0.05±0.10^{a}$	$0.1±0^{b}$	$0.2±0^{c}$	$1.3±0.22^{b}$	$0.2±0^{c}$
α-Terpinene	$0.042±0.004^{a}$	$0.04±0.09^{a}$	$0.1±0.01^{c}$	$0.5±0.15^{b}$	$0.1±0.01^{c}$	$0.5±0.1^{b}$
**Sesquiterpene hydrocarbons**						
γ-himachalane	$0.1±0^{a}$	$0.1±0.01^{a}$	$0.3±0.05^{b}$	$0.5±0.1^{c}$	$0.1±0.01^{a}$	$0.5±0.1^{c}$
Isolongifolene	$0.3±0.02^{a}$	$0.48±0.1^{b}$	$0.3±0.02^{a}$	$0.5±0.1^{b}$	$0.6±0.03^{c}$	$0.5±0.04^{b}$
β-Elemene	$0.50±0.03^{b}$	$0.2±0.94^{a}$	$0.5±0.04^{b}$	$0.78±0.08^{c}$	$0.4±0.04^{b}$	$0.77±0.07^{c}$
Zingiberene	$0.724±0.05^{a}$	$1±1.361^{ab}$	$0.8±0.04^{a}$	$1.5±0.09^{b}$	$1.4±0.09^{b}$	$1.5±0.06^{b}$
**Tunisia**						
Oil yield %	$6.9±0.1^{a}$	$9.5±0.44^{b}$	$8±0.1^{ab}$	$10.7±0.25^{b}$	$10±0.2^{b}$	$13.2±0.1^{c}$

Essential oil %	2.2±0^{a}	3.1±0.14^{b}	2.6±0 ^{a}	3.4±0.08^{b}	3.2±0.1^{b}	4.3±0.04^{c}
**Phenylpropanoids**						
trans-anethole	46.1±0.8^{a}	63.2±3.6^{b}	49.7±0.5^{a}	68.6±1.5^{b}	55.4±2.1^{a}	76.1±0.6^{b}
o-isoeugenol	2.8±0.1^{a}	4.7±0.35^{b}	2.9±0^{a}	4.8±0.22^{b}	3.2±0^{a}	5.2±0.2^{b}
p-anisaldehyde	0.1±0^{a}	0.25±0.01^{b}	0.3±0.01^{b}	0.4±0.02^{bc}	0.4±0^{bc}	0.5±0.01^{c}
Anisole	3±0.2^{a}	4.4±0.25^{b}	3.4±0.1^{a}	4.45±0.12^{b}	4.1±0.1^{b}	5.8±0.19^{c}
p-Anisaldehyde	0.2±0 ^{a}	0.2±0.01^{a}	0.7±0.2^{d}	0.307±0.05^{b}	3.5±0.1^{b}	5.2±0.23^{c}
Estragole	7.4±0.2^{a}	11±0.18^{bc}	7.6±0.1^{a}	10.1±0.64^{b}	9.7±0.1^{b}	12.7±0.5^{c}
**Monoterpene hydrocarbons**						
α-pinene	0.038±0.01^{a}	0.035±0.003^{a}	0.06±0^{b}	0.11±0.01^{c}	2.41±0.11^{d}	2.97±0.27^{d}
Limonene	0.2±0.01^{a}	0.4±0.02^{b}	0.3±0^{ab}	0.4±0.02^{b}	1.5±0.1^{c}	1.8±0.17^{c}
Myrcene	0.2±0.2^{a}	0.6±0.04^{b}	2.6±0.1^{d}	3.6±0.38^{e}	1.9±0.1^{c}	2.9±0.25^{d}
Linalool	2.2±0.1^{a}	2.6±0.27^{a}	2.2±0.1^{a}	2.8±0.18^{a}	2.6±0.1^{a}	3.9±0.3^{b}
Cis-β-ocimene	0.3±0.1^{a}	0.6±0.06^{b}	0.5±0^{b}	1±0.03^{c}	2.3±0.1^{c}	3±0.2^{c}
Sabinene	0.2±0^{a}	0.4±0.09^{b}	0.9±0.1^{c}	1±0.12^{c}	1±0.1^{c}	1.2±0.1^{c}
p-cymene	0.6±0^{a}	0.7±0.05^{a}	0.9±0.1^{b}	0.8±0.05^{b}	2.1±0^{c}	2.1±0.15^{c}
Aα-phellandrene	2±0.1^{a}	4±0.25^{b}	2.2±0.1^{a}	3.9±0.4^{b}	2.3±0.1^{a}	2±0.13^{a}
**Oxygenated monoterpenes**						
Fenchone	4.8±0.5^{b}	3.5±0.3^{a}	6.2±0.3^{bc}	5.4±0.4^{b}	7.3±0.4^{c}	6.3±0.5^{bc}
1,8-cineole	1.9±0.2^{a}	2.3±0.32^{a}	2.1±0.1^{a}	2.5±0.26^{a}	2.5±0.1^{a}	2.9±0.24^{b}
α-fenchyl acetate	0.099±0.09^{a}	0.099±0.002^{a}	0.177±0.17^{b}	0.2±0.01^{b}	1.5±0.3^{c}	2.2±0.53^{d}
α-Terpinene	0.091±0.09^{a}	0.091±0 ^{a}	0.189±0.20^{b}	0.2±0.01^{b}	0.1±^{a}0	0.2±0^{b}
**Sesquiterpene hydrocarbons**						
γ-himachalane	0.1±0^{a}	0.1±0.01^{a}	0.65±0.2^{b}	0.4±0.06^{bc}	0.1±0^{a}	0.2±0.02^{b}
Isolongifolene	0.3±0^{a}	0.3±0.03^{a}	0.47±0.3^{b}	0.4±0.03^{b}	0.7±0^{c}	0.8±0.12^{c}
β-Elemene	0.685±0.42^{ab}	0.685±0.08^{ab}	0.721±0.48^{b}	0.7±0.08^{b}	0.5±0^{a}	0.6±0.04^{ab}
Zingiberene	0.962±1.5^{a}	1.29±0.1^{a}	1.15±1.5^{a}	1.1±0.14^{a}	1.6±0.1^{ab}	2.1±0.47^{b}
			**Syria**			
Oil yield %	9±0.19^{a}	8.6±0.12^{a}	10.4±0.07^{ab}	9.3±0.14^{a}	12.4±0.4^{b}	10.6±0.1^{ab}
Essential oil %	2.9±0.06^{a}	2.8±0.04^{a}	3.3±0.02^{b}	3±0.05^{a}	4±0.14^{c}	3.4±0.05^{b}
**Phenylpropanoids**						
trans-anethole	59.9±1.08^{a}	53±0.14^{a}	66.3±1.02^{ab}	54±0.41^{a}	74±3.2^{b}	53.9±0.4^{a}
o-isoeugenol	5.2±0.3^{ab}	4.9±0.3^{a}	5.4±0.3^{ab}	4.6±0.3^{a}	5.8±0.43^{b}	5±0.39^{a}
p-anisaldehyde	0.1±0^{b}	0.1±0.01^{b}	0.4±0.05^{a}	0.4±0.04^{a}	0.5±0.05^{a}	0.5±0.03^{a}
Anisole	2.9±0.19^{a}	3.7±0.4^{b}	2.7±1.1^{a}	3.9±0.2^{b}	4±0.1^{b}	3.8±0.4^{b}
p-Anisaldehyde	0.2±0.01^{a}	0.2±0.01^{a}	2.0±0.9^{b}	3.157±0.^{c}	3.5±0.1^{c}	3.6±0.4^{c}
Estragole	11.2±0.7^{a}	12.4±1^{ab}	11.3±0.5^{a}	11.8±1^{a}	12±0.6^{ab}	13.8±0.9^{b}
**Monoterpene hydrocarbons**						
α-pinene	0.032±0.007^{a}	0.033±0.004^{a}	0.1±0.01^{b}	0.17±0.01^{c}	2.56±0.04^{d}	2.45±0.05^{d}

Limonene	0.3±0.05[a]	0.3±0.05[a]	0.4±0.06[b]	0.57±0.08[ab]	1.5±0.06[c]	1.4±0.1[c]
Myrcene	0.4±0.03[a]	0.5±0.04[a]	3.7±0.25[c]	3.65±0.2[c]	2.7±0.17[b]	2.5±0.05[b]
Linalool	3.5±0.23[a]	3.6±0.21[a]	3.6±0.2[a]	3.7±0.2[a]	3.5±0.23[a]	3.6±0.07[a]
Cis-β-ocimene	0.34±0.09[a]	0.4±0.1[a]	0.6±0.13[b]	0.7±0.13[b]	3.4±0.1[c]	3.8±0.2[c]
Sabinene	0.4±0.02[a]	0.4±0.02[a]	0.9±0.07[b]	0.8±0.03[b]	1±0.08[b]	1.1±0.03[b]
p-cymene	0.5±0.02[a]	0.7±0.03[ab]	0.5±0.02[a]	0.8±0.04[b]	1.7±0.06[c]	0.6±0.02[a]
Aα-phellandrene	3±0.2[bc]	2.7±0.17[b]	3.3±0.12[c]	2.6±0.1[b]	1.4±0.06[a]	2.1±0.1[ab]
**Oxygenated monoterpenes**						
Fenchone	3.9±0.25[a]	4±0.25[a]	5.4±0.07[b]	5.1±0.13[ab]	6.4±0.08[c]	4.5±0.12[a]
1,8-cineole	1.9±0.1[a]	2±0.12[a]	2.2±0.1[a]	2±0.11[a]	3.7±0.73[b]	2.9±0.11[ab]
α-fenchyl acetate	0.02±0.01[a]	0.05±0.05[b]	0.14±0.01[c]	0.3±0.02[d]	1.6±0.3[e]	1.3±0.02[e]
α-Terpinene	0.12±0.03[b]	0.043±0.05[a]	0.1±0.01[b]	0.3±0.03[c]	0.2±0.01[bc]	0.3±0.02[c]
**Sesquiterpene hydrocarbons**						
γ-himachalane	0.1±0.01[a]	0.2±0.01[ab]	0.4±0.07[b]	1±0.02[c]	0.1±0.01[a]	0.3±0.02[b]
Isolongifolene	0.3±0.01[a]	0.3±0.01[a]	0.4±0.02[a]	0.4±0.01[a]	0.7±0.11[b]	2.3±0.2[c]
β-Elemene	0.2±0.4[a]	0.60±0.67[b]	0.6±0.02[b]	1±0.08[c]	0.6±0.03[b]	0.7±0.02[bc]
Zingiberene	0.4±0.59[a]	0.741±0.8[b]	1±0.04[b]	1.3±0.2[c]	1.7±0.27[c]	2.7±0.1[d]
			**Turkey**			
Oil yield %	7.6±0.22[a]	9.3±0.2[b]	8.8±0.17[ab]	10.2±0.08[b]	11.2±0.3[b]	12.2±0.3[b]
Essential oil %	2.5±0.07[a]	3±0.08[b]	2.8±0.05[ab]	3.3±0.03[b]	3.6±0.13[bc]	3.9±0.12[c]
**Phenylpropanoids**						
trans-anethole	51.5±1.4[a]	66.2±2.8[b]	56.3±1.2[a]	71±1.1[bc]	62.7±2.5[b]	79±1.93[c]
o-isoeugenol	3.9±0.27[a]	3.9±0.26[a]	4.1±0.29[a]	4±0.35[a]	4.4±0.31[a]	4.3±0.38[a]
p-anisaldehyde	0.1±0[a]	0.1±0 [a]	0.3±0.02[b]	0.3±0.03[b]	0.4±0.02[b]	0.4±0.0[b]
Anisole	3.5±0.12[b]	2.8±0.18[ab]	3.4±0.1[b]	1.94±0.6[a]	4.6±0.22[c]	2.8±0.38[ab]
p-Anisaldehyde	0.2±0.01[a]	0.2±0.01[a]	0.71±0.15[c]	0.4±0.2[b]	6.6±0.6[e]	3.4±0.35[d]
Estragole	8.6±0.17[a]	11.6±0.4[ab]	8.9±0.18[a]	10.5±0.4[ab]	9.5±0.7[a]	12.3±0.3[b]
**Monoterpene hydrocarbons**						
α-pinene	0.032±0.004[a]	0.031±0.003[a]	0.09±0.01[b]	0.09±0[b]	1.3±0.14[c]	2.12±0.11[d]
Limonene	0.3±0.02[a]	0.3±0.03[a]	0.4±0.02[a]	0.3±0.03[a]	1.4±0.1[b]	1.3±0.05[b]
Myrcene	0.4±0.02[a]	0.4±0.02[a]	2.8±0.14[bc]	3.2±0.31[c]	2.1±0.11[b]	2.4±0.22[b]
Linalool	2.1±0.05[a]	2.4±0.18[a]	2.3±0.06[a]	2.6±0.13[a]	2.8±0.15[ab]	3.1±0.29[b]
Cis-β-ocimene	0.3±0.03[a]	0.3±0.02[a]	0.6±0.04[b]	0.5±0.05[b]	2.4±0.06[c]	2.7±0.13[c]
Sabinene	0.4±0.01[a]	0.4±0.04[a]	1±0.08[b]	1±0.09[b]	1.1±0.09[b]	1.1±0.1[b]
p-cymene	0.6±0.03[a]	0.6±0.02[a]	0.6±0.03[a]	0.6±0.02[a]	1.9±0.08[b]	1.9±0.08[b]
Aα-phellandrene	2.9±0.09[b]	3.1±0.24[b]	3.3±0.08[b]	3.4±0.17	1.7±0.07[b]	1.7±0.05[a]
**Oxygenated monoterpenes**						
Fenchone	2.3±0.15[a]	3.2±0.3[b]	3.8±0.1[b]	3.8±0.1[b]	3.9±0.1[b]	3.4±0.18[b]
1,8-cineole	1.7±0.24[a]	1.6±0.1[a]	1.9±0.15[a]	1.8±0.08[a]	4.8±1.4[b]	2.1±0.12[a]

α-fenchyl acetate	0.05±0.0^{b}	0.020±0.07^{a}	0.4±0.1^{d}	0.1±0^{c}	1.8±0.1^{e}	1.4±0.3^{e}
α-Terpinene	0.043±0.0^{b}	0.01±0.09^{a}	0.1±0^{b}	0.1±0.01^{b}	0.8±0.3^{d}	0.3±0.05^{c}
**Sesquiterpene hydrocarbons**						
γ-himachalane	0.1±0.01^{a}	0.1±0.01^{a}	0.3±0.06^{b}	0.3±0.04^{b}	0.2±0.03^{ab}	0.1±0.01^{a}
Isolongifolene	0.3±0.02^{b}	0.2±0^{a}	0.4±0.01^{a}	0.2±0^{a}	1±0.08^{c}	0.6±0.08^{b}
β-Elemene	0.58±0.03^{b}	0.2±0.85^{a}	0.5±0.04^{c}	0.5±0.03^{c}	0.5±0^{c}	0.4±0.01^{b}
Zingiberene	0.73±0.04^{b}	0.2±0.74^{a}	0.9±0.06^{bc}	0.8±0.03^{b}	1.5±0.15^{c}	1.4±0.27^{c}
**Yemen**						
Oil yield %	12.4±0.15^{a}	12.8±0.2^{a}	14.2±0.17^{ab}	13.1±0.17^{ab}	16.3±0.39^{b}	14±0.16^{ab}
Essential oil %	4±0.05	4.1±0.07	4.6±0.06	4.2±0.06	5.3±0.13	4.5±0.05
**Phenylpropanoids**						
trans-anethole	80.5±1.2^{a}	90.4±2.2^{b}	88.4±1.5^{b}	89.4±2.1^{b}	88.3±1.4^{b}	89.3±2^{b}
o-isoeugenol	6.5±0.13^{ab}	5.8±0.11^{a}	6.7±0.09^{ab}	5.5±0.1^{a}	7.3±0.09^{b}	5.8±0.11^{a}
p-anisaldehyde	0.1±0.01^{a}	0.1±0^{a}	0.5±0.01^{c}	0.3±0.01^{ab}	0.7±0.02^{d}	0.4±0.01^{b}
Anisole	5.9±0.33^{a}	5±0.2^{a}	5.862±0.7^{a}	5.095±0.222^{a}	7.6±0.53^{b}	5±0.19^{a}
p-Anisaldehyde	0.3±0.01^{a}	0.3±0.01^{a}	0.331±0.04^{a}	0.32±0.008^{a}	6.9±0.5^{c}	4.8±0.18^{b}
Estragole	15.2±0.35^{a}	15.5±0.3^{a}	14±0.86^{a}	14.6±0.28^{a}	13.5±1.56^{a}	15.9±0.3^{a}
**Monoterpene hydrocarbons**						
α-pinene	0.053±0.002^{a}	0.05±0.003^{a}	0.19±0.01^{b}	0.14±0^{b}	1.64±0.05^{c}	3.98±0.05^{d}
Limonene	0.44±0.02^{b}	0.2±0.01^{a}	0.6±0.03	0.3±0.01^{ab}	2.4±0.05^{d}	0.9±0.06^{c}
Myrcene	0.8±0.05^{b}	0.6±0.02^{a}	5.8±0.3f	3.8±0.09^{d}	4.2±0.2^{e}	2.8±0.06^{c}
Linalool	4.6±0.05^{b}	3.5±0.13^{a}	4.7±0.05^{b}	3.8±0.09^{a}	5.6±0.29^{e}	3.7±0.08^{a}
Cis-β-ocimene	0.5±0.08^{a}	0.5±0.02^{a}	0.9±0.09^{b}	0.6±0.02^{a}	4.9±0.05^{c}	3.9±0.09^{d}
Sabinene	0.6±0.01^{a}	0.6±0.03^{a}	1.6±0.13^{b}	1±0.01^{ab}	1.8±0.15^{b}	1.2±0.01^{ab}
p-cymene	1±0.03^{a}	1±0^{a}	1.1±0.04^{a}	1±0.01^{a}	3.2±0.15^{c}	0.6±0.01^{b}
Aα-phellandrene	4.4±0.04^{b}	4.7±0.2^{b}	5±0.18^{b}	4.4±0.2^{b}	2.9±0.11^{a}	2.6±0.02^{a}
**Oxygenated monoterpenes**						
Fenchone	4.6±0.16^{a}	4.1±0.24^{a}	6.1±0.3^{b}	5.1±0.17^{b}	6.5±0.74^{b}	4.4±0.14^{a}
1,8-cineole	2.9±0.17^{b}	1.3±0.05^{a}	3.4±0.08^{bc}	1.3±0.05^{a}	4.1±0.12^{c}	1.6±0.05^{a}
α-fenchyl acetate	0.101±0.09^{a}	0.089±0.0^{a}	0.3±0.01^{bc}	0.2±0^{b}	2.7±0.54^{bc}	0.4±0^{c}
α-Terpinene	0.093±0.12^{a}	0.103±0.0^{a}	0.3±0.02^{bc}	0.2±0^{b}	0.4±0.02^{c}	0.2±0^{b}
**Sesquiterpene hydrocarbons**						
γ-himachalene	0.2±0^{b}	0.1±0.01^{a}	0.6±0.09^{c}	0.2±0^{b}	0.3±0^{b}	0.2±0^{b}
Isolongifolene	0.5±0.02^{a}	0.38±0.01^{a}	0.6±0.02^{b}	0.5±0.01^{b}	1.5±0.12	0.7±0.02^{b}
β-Elemene	0.36±0.72^{a}	0.647±0.08^{b}	1.2±0.01^{c}	0.8±0.02^{b}	1±0.04^{c}	0.7±0.01^{b}
Zingiberene	0.64±1.5^{a}	0.99±0.17^{ab}	1.9±0.19^{b}	1.2±0.04^{ab}	3.5±0.44^{c}	1.8±0.05^{b}
**Morocco**						
Oil yield %	6.7±0.1^{a}	7.8±0.1^{a}	7±0.2^{a}	8±0.1^{ab}	7.9±0.2^{a}	8.8±0.1^{b}
Essential oil %	2.1±0^{a}	2.5±0.0^{a}3	2.3±0.1^{a}	2.6±0.03^{a}	2.5±0.1^{a}	2.8±0.04^{a}

**Phenylpropanoids**						
trans-anethole	43.7±1^{a}	55.9±0.91^{b}	43.7±1^{a}	55.5±0.8^{b}	43.7±1^{a}	55.4±0.8^{b}
o-isoeugenol	3.4±0.2^{a}	3.8±0.1^{a}	3.2±0.2^{a}	3.6±0.1^{a}	3.4±0.2^{a}	3.8±0.1^{a}
p-anisaldehyde	0.04±0^{a}	0.1±0.0^{b}	0.2±0^{c}	0.2±0.0^{c}	0.3±0^{d}	0.3±0.0^{d}
Anisole	2.7±0.1^{a}	3.2±0.1^{a}	2.6±0.1^{a}	3±0.1^{b}	2.8±0.1^{a}	3.2±0.1^{a}
p-Anisaldehyde	0.2±0^{a}	0.2±0.012^{a}	0.2±0^{a}	0.2±0.01^{a}	2.7±0.1^{ab}	3.1±0.0^{b}
Estragole	8.2±0.3	9.9±0.177	7.8±0.2	9.3±0.163	9.3±0.3	10.2±0.187
**Monoterpene hydrocarbons**						
α-pinene	0.043±0.002^{a}	0.04±0.003^{a}	0.14±0.01^{b}	0.18±0^{b}	1.01±0.05^{c}	1.98±0.05^{d}
Limonene	0.2±0^{a}	0.2±0.0^{a}	0.3±0^{b}	0.3±0.03^{b}	1±0.1^{c}	0.7±0.07^{c}
Myrcene	0.4±0^{a}	0.4±0.01^{a}	2.7±0.1^{ab}	2±0.03^{b}	1.9±0.^{b}	1.5±0.00^{ab}
Linalool	2.6±0.1^{b}	1.8±0.014^{a}	2.7±0.1^{b}	1.8±0.08^{a}	3.2±0.3^{bc}	2±0.008^{a}
Cis-β-ocimene	0.3±0^{a}	0.3±0.0^{a}	0.4±0^{b}	0.5±0.02^{b}	4.8±0.6^{d}	1.9±0.08^{c}
Sabinene	0.3±0^{a}	0.4±0.02^{ab}	0.5±0^{b}	0.4±0.009^{ab}	0.9±0.2^{c}	0.5±0.046^{b}
p-cymene	0.5±0^{b}	0.4±0.01^{ab}	0.5±0^{b}	0.4±0.01^{ab}	0.3±0^{a}	1.5±0.04^{c}
Aα-phellandrene	2.6±0.1^{b}	1.7±0.054^{ab}	2.5±0.1^{b}	1.6±0.049^{ab}	1.3±0.1^{a}	1±0.03^{a}
**Oxygenated monoterpenes**						
Fenchone	2.5±0.1^{a}	2.8±0.12^{a}	3.7±0.1^{b}	3.4±0.08^{ab}	3.1±0.1^{a}	2.9±0.07^{a}
1,8-cineole	1.5±0.1^{b}	0.8±0.05^{a}	1.5±0.1	0.8±0.04^{a}	1.6±0.1^{b}	0.9±0.04^{a}
α-fenchyl acetate	0.1±0^{a}	0.1±0.002^{a}	0.1±0^{a}	0.1±0.002^{a}	0.3±0^{b}	0.9±0.01^{c}
α-Terpinene	0.1±0^{a}	0.1±0.001^{a}	0.14±0^{a}	0.1±0^{a}	0.1±0^{a}	0.4±0.004^{b}
**Sesquiterpene hydrocarbons**						
γ-himachalane	0.12±0.01^{a}	0.14±0.00^{a}2	0.1±0^{a}	0.13±0.001^{a}	0.17±0^{a}	0.15±0.00^{a}
Isolongifolene	0.2±0^{a}	0.3±0.007^{b}	0.3±0^{b}	0.3±0.005^{b}	0.4±0^{bc}	0.3±0.008^{b}
β-Elemene	0.4±0^{b}	0.25±0.013^{a}	0.4±0^{b}	0.4±0.01^{b}	0.4±0^{b}	0.4±0.0^{b}
Zingiberene	0.8±0^{a}	0.8±0.034^{a}	0.8±0^{a}	0.8±0.03^{a}	1±0^{a}	0.8±0.027^{a}
